# *Schistosoma haematobium* infection is associated with alterations in energy and purine-related metabolism in preschool-aged children

**DOI:** 10.1371/journal.pntd.0008866

**Published:** 2020-12-14

**Authors:** Derick N. M. Osakunor, Takafira Mduluza, Douglas Osei-Hyiaman, Karl Burgess, Mark E. J. Woolhouse, Francisca Mutapi

**Affiliations:** 1 Institute of Immunology & Infection Research, University of Edinburgh, Ashworth Laboratories, King’s Buildings, Edinburgh, United Kingdom; 2 Biochemistry Department, University of Zimbabwe, Mount Pleasant, Harare, Zimbabwe; 3 Laboratory of Physiologic Studies, National Institute on Alcohol Abuse and Alcoholism, National Institutes of Health, Bethesda, Maryland, United States of America; 4 Metabolomics Research Division, Human Metabolome Technologies Inc., Tsuruoka, Yamagata, Japan; 5 Department of Systems Neurophysiology, Graduate School of Medical & Dental Science, Tokyo Medical and Dental University, Bunkyo City, Tokyo, Japan; 6 Centre for Synthetic and Systems Biology, University of Edinburgh, CH Waddington Building, King’s Buildings, Edinburgh, United Kingdom; 7 Usher Institute, University of Edinburgh, Ashworth Laboratories, King’s Buildings, Edinburgh, United Kingdom; 8 NIHR Global Health Research Unit Tackling Infections to Benefit Africa (TIBA), University of Edinburgh, Ashworth Laboratories, King’s Buildings, Edinburgh, United Kingdom; Walter and Eliza Hall Institute of Medical Research, AUSTRALIA

## Abstract

Helminths are parasitic worms that infect over a billion people worldwide. The pathological consequences from infection are due in part, to parasite-induced changes in host metabolic pathways. Here, we analyse the changes in host metabolic profiles, in response to the first *Schistosoma haematobium* infection and treatment in Zimbabwean children. A cohort of 83 schistosome-negative children (2–5 years old) as determined by parasitological examination, guardian interviews and examination of medical records, was recruited at baseline. Children were followed up after three months for parasitological diagnosis of their first *S*. *haematobium* infection, by detection of parasite eggs excreted in urine. Children positive for infection were treated with the antihelminthic drug praziquantel, and treatment efficacy checked three months after treatment. Blood samples were taken at each time point, and capillary electrophoresis mass spectrometry in conjunction with multivariate analysis were used to compare the change in serum metabolite profiles in schistosome-infected versus uninfected children. Following baseline at the three-month follow up, 11 children had become infected with *S*. *haematobium* (incidence = 13.3%). Our results showed that infection with *S*. *haematobium* was associated with significant increases (>2-fold) in discriminatory metabolites, linked primarily with energy (G6P, 3-PG, AMP, ADP) and purine (AMP, ADP) metabolism. These observed changes were commensurate with schistosome infection intensity, and levels of the affected metabolites were reduced following treatment, albeit not significantly. This study demonstrates that early infection with *S*. *haematobium* is associated with alterations in host energy and purine metabolism. Taken together, these changes are consistent with parasite-related clinical manifestations of malnutrition, poor growth and poor physical and cognitive performance observed in schistosome-infected children.

## Author summary

Schistosomiasis is a parasitic disease caused by helminth worms called schistosomes, and millions of people worldwide are infected. Studies in animal models indicate that the infection can cause alterations in metabolism of the host, leading to the observed clinical manifestations of the disease. However, there are limited human studies on the impact of schistosome infection on host metabolism, and none describing the changes that occur early in infection. There are also no studies relating to the species *Schistosoma haematobium*, which causes disease manifested in both the urinary and genital organs. To address this, we analysed the metabolic changes in response to the first *S*. *haematobium* infection and treatment in Zimbabwean preschool-aged children. We determined for the first time, that within three months of first infection, there are significant changes in metabolite profiles related to host energy (G6P, 3-PG, AMP, ADP) and purine (AMP, ADP) metabolism. These changes were associated with infection burden and were resolved within three months, following curative treatment. Our findings provide further understanding into the early host metabolic responses to the infection, consistent with clinical manifestations such as malnutrition, poor growth and poor physical and cognitive performance observed in schistosome-infected children. This demonstrates the need for early treatment of the infection, and will inform the development of appropriate interventions such as nutraceuticals in child feeding programs, aimed at reducing disease consequences. Further mechanistic studies will contribute to more understanding of the relationship between metabolic alterations and schistosomiasis in young children.

## Introduction

Helminths are multicellular parasitic worms that infect over a billion people worldwide [1]. In tropical and sub-tropical regions with limited access to safe water and adequate sanitation provision, diseases caused by helminth infections including soil-transmitted helminths (STH) [1] and schistosomes are highly prevalent [2–4]. With an estimated 250 million people infected worldwide, schistosomiasis is a parasitic disease caused by helminth trematodes of the *Schistosoma* genus [5]; at least 90% of all cases occur in sub-Saharan Africa [6,7]. The species *Schistosoma haematobium*, accounts for about two-thirds of all schistosomiasis cases in Africa, and causes the urogenital form of the disease [8,9]. Diagnosis is typically by microscopic detection of eggs in urine, and treatment is by administration of the anti-helminthic drug, praziquantel [9].

In endemic areas with high infection transmission, infection with schistosomes is cumulative, and can begin in the first year of birth [10]. The health impacts of *S*. *haematobium* infection thus begin at this early age and can include haematuria, protein wasting [11–13], malnutrition, poor growth, and poor physical and cognitive performance [14]. Left untreated, infection can lead to chronic disease and pathology, including anaemia, poor reproductive health, increased susceptibility to sexually transmitted infections [14], prostate cancer [15], urothelial carcinogenesis [16], bladder dysfunction, fibrosis, and renal failure [17].

Interaction between the schistosome worm and its host is central to parasite survival. Successful parasitic relationships can thus be achieved by manipulating the host’s metabolism to divert essential nutrients and metabolites towards parasite growth. For instance, schistosome worms rely on host glucose as their main source of energy for survival [18,19]. In addition, schistosome infection induces parasite-specific immune responses that cause a downregulation and immuno-modulation of the host’s immune system, to promote parasite survival for decades [20–23]; the alteration in host immune responses can alter host metabolic function, disease patterns and overall host health [24]. Experimental schistosome studies have shown that in order to establish themselves and survive in the host (including growth, development and egg laying), schistosome worms require host-derived endocrine steroid and thyroid hormones [25–27], as well as immune molecules including tumour necrosis factor alpha [28,29], interleukin-7 [25], and CD4+ T lymphocytes [30]. When such host factors are unavailable to the parasite, there is poor parasite development and fecundity, and infection causes reduced pathology in the host [31–33]. Taken together, the available experimental evidence indicates that the alterations in systemic host pathways extend to metabolism. Current evidence from mouse models of schistosomiasis show that schistosome infection and disease are linked to alterations in gut microbiota metabolism [19,34], as well as changes in amino acid, lipid and energy metabolism [19,34–36]. Alterations in liver metabolism due to parasite egg-induced inflammatory responses have also been reported [37]. Studies characterising the metabolic changes to human schistosome infection are limited. However, analysis of urine samples has been recently applied to study the host metabolic changes in *S*. *mansoni*-infected children and adults [38,39]. The findings were similar to those from experimental models in terms of alterations in energy, liver, and gut microbiota metabolism, all of which are linked to morbidity from the infection [38,39].

While the significant detrimental effects of schistosome infections on host health are unarguable, some experimental and human epidemiological studies have suggested that the sustained host metabolic alterations from schistosome infections may reduce the occurrence and severity of other conditions, including metabolic syndrome [19,40–43]. The exact mechanisms of these effects remain to be fully understood [23]. It is clear that there is a need for further studies on the dynamics of host-parasite relations at the molecular level, to elucidate pathways involved in pathology and disease progression versus those ameliorating metabolic syndromes.

In the case of *S*. *haematobium* infection in infants and young children, little is known about the basic mechanisms underlying the pathophysiology of the disease [44]. There are limited human studies on the impact of schistosome infection on host metabolism, and metabolic phenotyping of blood samples from cases of human schistosomiasis has not been reported. The majority of previous investigations both in human and animal models have been based on well-established late stage schistosome infections; this does not allow the early metabolic changes associated with the first schistosome infection to be elucidated. The currently available evidence have also focused on *S*. *mansoni* and *S*. *japonicum* infections, and there have not yet been any published studies on the impact of human *S*. *haematobium* infection, on the host metabolic phenotype. Thus, this study aims to characterise the host metabolite profiles of Zimbabwean preschool-aged children (≤5 years) before their first *S*. *haematobium* infection, and the changes that occur following infection and treatment. It further determines the impact of such specific metabolite alterations on host metabolism and the development of schistosome-related morbidity. We hypothesised that early in the first *S*. *haematobium* infection in preschool-aged children, there are alterations in host metabolite profiles linked with metabolic pathways implicated in schistosome-related morbidity.

## Methods

### Ethics statement

Ethical and institutional approval for the main study (of which this is a subset) was obtained from the Medical Research Council of Zimbabwe (MRCZ/A/1964) and the University of Edinburgh (fmutapi-0002), respectively. The Medical Director of the Mashonaland Central Province also granted permission to conduct the study in the area. The study aims and procedures were explained to participants’ parents/guardians in Shona, the local language, and participation in the study was voluntary. Written informed consent was obtained from the participants’ parents/guardians.

### Study design, population and site

The study followed a longitudinal design, embedded within a larger paediatric urogenital schistosomiasis study conducted in the Shamva district, Northeast Zimbabwe. The study was designed to detect the first schistosome infection in preschool-aged children, and to determine the health impact of infection and treatment as previously published [13]. The study area, Madziwa (17°04′S 31°40′E), was chosen because a previous national survey showed that the area has a high prevalence of *S*. *haematobium* (>50%), and a low prevalence of *S*. *mansoni* and STH (<15%) [45].

**Fig 1:**
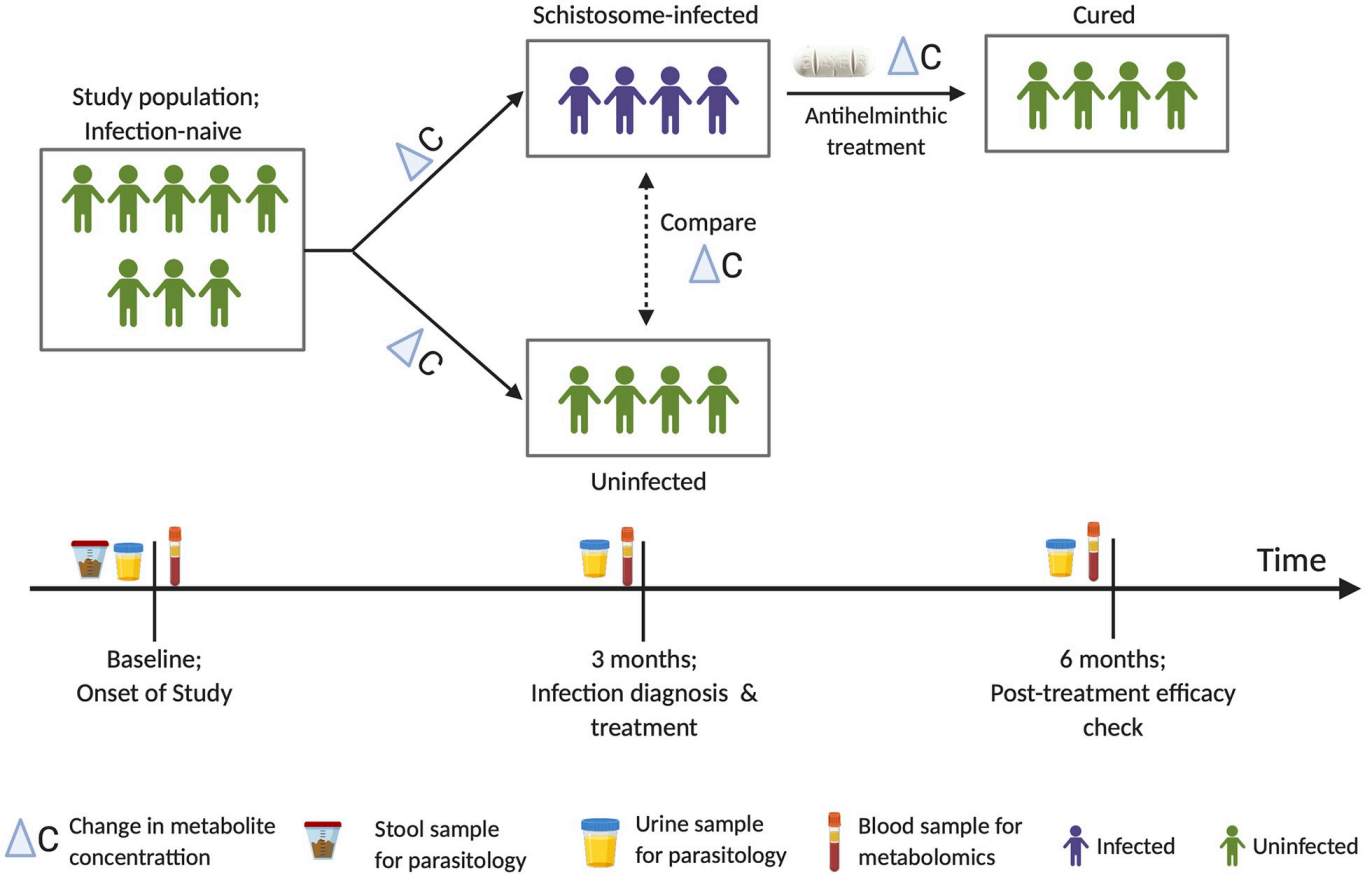
Summary of study design. Schistosome-infected and uninfected refer to diagnosis by microscopic egg counts in urine.

While in experimental studies it is possible to infect animals at specific time points, follow the course of infection, and investigate its impact on the host, this is not possible in human hosts. Hence in the current study, we followed natural infection of populations living in schistosome-endemic areas. The study included 83 children, aged 2–5 years, not previously infected or treated for schistosomiasis (assessed by health questionnaire and clinical records) and confirmed schistosome negative (by egg count for *S*. *haematobium* and *S*. *mansoni*). The children were also diagnosed for other helminths to exclude STH infections via stool examination (by egg count). At baseline, a questionnaire was administered at recruitment to gather metadata on basic socio-demography and anthropometric measurements including height, weight, and mid-upper arm circumference (MUAC) as previously described [13]. A venous blood sample was also collected from each child and later processed to collect serum for metabolite analysis. Children were then followed up three months later to detect their first *S*. *haematobium* infection (by egg count). At this follow up survey, a second blood sample was collected from all children for analysis to detect changes in metabolite profiles from baseline. Both experimental and previous field studies show that in three months, any new *S*. *haematobium* infections acquired would have reached patency and thus be detectable by egg excretion in urine [46–48]. All children who were positive for *S*. *haematobium* infection were treated by clinical staff (local nurses from the Madziwa rural clinic) with a single dose of praziquantel at the standard 40 mg/kg body weight as previously described [4]. For treated children, a post-treatment efficacy check (by egg count) and blood sampling was carried out three months later. Fig 1 shows a summary of the study design.

### Sample collection and processing

#### Parasitology samples

For schistosomiasis diagnosis, approximately 50 mL of urine was collected on three consecutive days at each time point, and a stool specimen was collected on a single day at baseline. Urine samples were examined microscopically for *S*. *haematobium* infection following the standard urine filtration procedure [49], and infection intensity was reported as the arithmetic mean number of eggs per 10 mL urine of at least two urine samples. The stool samples collected were processed using the Kato–Katz method (in duplicates) [50], and parasite eggs enumerated microscopically to exclude *S*. *mansoni* and STH. Children were positive for infection if at least one parasite egg was detected in at least one of the urine or stool samples.

#### Blood samples

Up to 5 mL of venous blood was collected from each participant, allowed to clot at room temperature for about 30 minutes, and stored at 4°C for a maximum of 4 hours. Serum was obtained from the samples after centrifugation at 3000 rpm for 10 minutes, frozen at -20°C in the field and transferred to a −80°C freezer in the laboratory (University of Zimbabwe), prior to cold-chain shipment to the University of Edinburgh, UK. For long-term storage, samples were kept at −80°C until shipped on dry ice to Human Metabolome Technologies Inc. (HMT; Yamagata, Japan) for metabolite analysis. Appropriate pre-analytical considerations for processing blood samples for metabolite analysis were followed [51]. Blood samples were collected rather than urine samples for metabolite analysis because blood is less susceptible to metabolite variations related to sample collection time, meal intake and hydration levels [52]. To minimise the effects of meal and sample-time factors on metabolite analysis, non-fasting pre-meal samples were collected at about midday during recruitment [52].

#### Metabolite measurements: CE–TOF–MS

Sample preparation and metabolite analysis was carried out by HMT using the capillary electrophoresis time-of-flight mass spectrometry (CE–TOF–MS)-based metabolomics technique [53,54]. 50 μL of serum sample was mixed with 450 μL of methanol containing 10 μM internal standards. Chloroform (500 μL) and Milli-Q water (200 μL) were added, mixed thoroughly and centrifuged (2,300 x g, 4°C for 5 minutes). The water layer (400 μL) was filtered through a 5-kDa cut-off filter (ULTRAFREE-MC-PLHCC; HMT, Yamagata, Japan) to remove macromolecules. The filtrate was concentrated by centrifugation and resuspended in 50 μL of ultrapure water immediately before measurement. Spectra profiles were obtained using a CE–TOF–MS (Agilent Technologies Inc. Waldbronn, Germany) system. Full details of experimental conditions and instrumentation are as previously described [55–57] (details described in S1 Text).

#### CE-TOF-MS data acquisition and processing

Peak information including mass-to-charge ratio (*m/z*), migration time (MT), and peak area, were extracted using automatic integration software (MasterHands ver. 2.17.1.11 developed at Keio University). Relative peak area was calculated using a peak detection limit based on signal-noise ratio (S/N) = 3 [57]:

$$Relative\;Peak\;Area\;=\;\frac{Metabolite\;Peak\;Area}{Internal\;Standard\;Peak\;Area\times Sample\;Amount}$$ (1)

For peak annotation, putative metabolites were then assigned from HMT’s standard library and Known-Unknown peak library based on *m/z* and MT. In instances where a feature matched with multiple annotations within their m/z and MT windows, all alternatives are provided. The tolerance level was ±0.5 min in MT and ±10 ppm in *m/z* [56].

Masserror(ppm)=MeasuredValue-TheoreticalValueMeasuredValue×106 (2)

Using standard curves obtained by single-point (100 μM) calibrations, absolute metabolite concentrations were calculated by normalizing the peak area of each metabolite with respect to the area of the internal standard. This minimised technical variability and also enabled sample to sample comparisons in data analysis.

A total of 248 metabolite peaks (145 in cation and 103 in anion mode respectively) were detected and annotated based on HMT’s standard and Known-Unknown peak library. Of these, 70 target metabolites were detected and quantified (40 in cation and 30 in anion mode respectively), and these were used for all downstream analysis. Individual samples for which a target peak or metabolite was below detection limits and thus could not be quantified, the peak area or concentration of the metabolite was captured as “Not detected (N.D)”.

### Data analyses

Data analyses and visualisations were performed using SPSS version 22 (IBM Corp.), GraphPad Prism version 8.2.0 (GraphPad Software, Inc), and MetaboAnalyst, a web-based tool for the analysis of metabolomic data [58–60]. Continuous data are presented as mean (standard deviation; SD) or as median (interquartile range; IQR). Categorical data are presented as absolute numbers and percentages.

Prior to analysis, a data integrity check was performed (MetaboAnalyst), and missing values (in this case “N.D”) caused by metabolites below the detection limit were replaced by a small value (i.e. half the minimum positive value in the data set = 0.15 μM), as per standard practice in MetaboAnalyst (details in S1 Text). For changes in metabolite concentrations in response to schistosome infection, the change in metabolite concentrations (ΔC) at baseline (C1) and at follow up (C2) for infection was calculated as ΔC μM = C2 –C1 (μM). By default, MetaboAnalyst removes data for metabolites with a constant or a single value across samples. For metabolite analysis at baseline, six metabolites were found and removed. Likewise, to improve statistical power for metabolite analysis of the change in metabolite profile data, eight additional metabolites with less than n = 10 non-zero change in concentration values (ΔC) across samples were excluded from analysis with the change in concentration data set (14 metabolites in total) [details in S1 Text]. For all analyses, data were processed by range scaling [61].

To determine if the mean differences in metabolite profiles between groups of interest were likely due to chance, Multivariate Analysis of Variance (MANOVA; SPSS) with sequential sums of squares was used, as recommended for pathogen-related studies [62]. The model to determine and account for underlying age and sex-related effects at baseline included age (years), sex, and their interaction, in that order. To determine the change in metabolite concentration due to schistosome infection, a model including age (years), sex, infection status and their interactions, in that order was used. Where the variable of interest was found to be significant, the model was then re-run without the significant variable, and the residuals from the resulting model were saved and subjected to further analysis to identify discriminatory metabolites compared across that variable (MetaboAnalyst). This was to ensure that the confounding effects of other factors such as age and sex were already accounted for, prior to downstream analysis to determine the most relevant metabolites accounting for differences in metabolite profiles between groups of interest.

Residuals from metabolite concentration data (from MANOVA models) along with participant metadata were imported into MetaboAnalyst. Univariate analysis using fold change (FC) and pattern correlation analysis (Pearson’s) were used to identify metabolites that are potentially significant in discriminating between two groups, and to show metabolite patterns of change under different conditions. A false discovery rate (FDR) threshold of <0.05 [63] and a concentration ratio (i.e. between two groups) of at least 2-fold was considered significant [64]. For an informative first-hand look at the data set, an unsupervised Principal Component Analysis (PCA) was employed to assess clustering trends and group separation in the data set. To identify specific metabolites accounting for differences in metabolite profiles between groups of interest, a supervised multiple regression analysis method, Orthogonal Projections to Latent Structures Discriminant Analysis (OPLSDA) [65] with Orthogonal Signal Correction (OSC) filtering [66], was used to discriminate groups and identify the differentially expressed metabolites that drive group separation. This supervised method has been shown to be more reliable at overcoming the limitations of heterogeneity associated with analysis of human metabolomic data, where PCA does not reveal changes in metabolite profiles across groups [39,67]. Cumulative model statistics, R^2^Y (cum) and Q^2^ (cum), were calculated for each model and used to assess the degree of fit and predictive reliability respectively [68]. The significance of the model was evaluated using permutation testing (n = 1000), with a p-value threshold of 0.05 [69]. For all valid OPLSDA models, a combination of a generated S-plot [absolute p(corr) >0.5] and the variable importance in the projection (VIP) values (VIP ≥1.5) were used to identify and select significant differentially expressed metabolites between groups [70]. To further determine the relationship between significant differentially expressed metabolites (identified from the *S*. *haematobium* infection status OPLSDA model) and infection intensity, range-scaled residuals from the change in concentration metabolite data set (from MANOVA) were regressed on the log-transformed infection intensity (log_10_ [egg count+1]).

To identify metabolic pathways associated with schistosome infection and facilitate further biological interpretation, metabolite pathway analysis was performed in MetaboAnalyst; this combines results from powerful pathway enrichment analysis with pathway topology analysis. Data for the significant differentially expressed metabolites identified from the infection status OPLSDA model were queried against associated *Homo sapien*s metabolic pathway libraries (downloaded on 04.06.2019), curated from Kyoto Encyclopedia of Genes and Genomes (KEGG; http://www.genome.jp/kegg/). Full details of analysis are in S1 Text.

## Results

### Population characteristics

As shown in Table 1, median age was 3 years (range; 2–5 years) and 41 (49.4%) of the children were female. At follow up, 11 (13.3%) were positive for *S*. *haematobium* infection with mean infection intensity of 0.8 (SEM = 0.3; 95% CI = 0.2–1.5) eggs/10 mL of urine.

**Table 1:** Participant characteristics.

Variable	Total	Female	Male
	Baseline		
**Age (years)**			
Median	3 (3–4)	3 (3–4)	3.5 (3–4.3)
2	8 (9.6)	4 (9.8)	4 (9.5)
3	35 (42.2)	18 (43.9)	17 (40.5)
4	25 (30.1)	14 (34.1)	11 (26.2)
5	15 (18.1)	5 (12.2)	10 (23.8)
**Height (cm)**	96.0 (91.0–102.0)	96.0 (91.5–98.0)	100.0 (91.0–104.0)
**Weight (kg)**	14.0 (12.7–16.0)	14.0 (12.1–15.0)	15.0 (13.0–16.0)
**MUAC (cm)**	15.0 (14.0–16.0)	14.6 (14.0–15.4)	15.0 (14.0–16.0)
**Mean WHZ**	-0.29 (1.29)	-0.30 (1.23)	-0.28 (1.36)
**Mean WAZ**	-0.59 (1.19)	-0.62 (1.23)	-0.56 (1.16)
**Mean HAZ**	-0.70 (1.38)	-0.69 (1.45)	-0.71 (1.33)
	**Follow up (3 months)**		
***S*. *h* Infection status**			
Negative	72 (86.7)	34 (82.9)	38 (90.5)
Positive	11 (13.3)	7 (17.1)	4 (9.5)
***S*. *h* Infection intensity (eggs/10 mL urine)**	0.8 (0.2–1.5)	0.5 (0.1–1.0)	1.1 (0–2.3)

Table shows the characteristics of the sample population at baseline and at follow up. Growth and nutritional indices adjusted for age and expressed as Z-scores [71] was calculated using the WHO Anthro software (version 3.0.1; http://www.who.int/childgrowth/en/). Data are expressed as median (IQR) or n (%), except for WHZ, WAZ, and HAZ, which are mean (SD). *S*. *haematobium* infection intensity is shown as mean (95% confidence interval). S. h., *S*. *haematobium*; WHZ, weight–for-height Z-scores; WAZ, weight–for-age Z-scores HAZ, height-for-age Z scores.

### Baseline metabolic differences associated with age and sex

**Fig 2:**
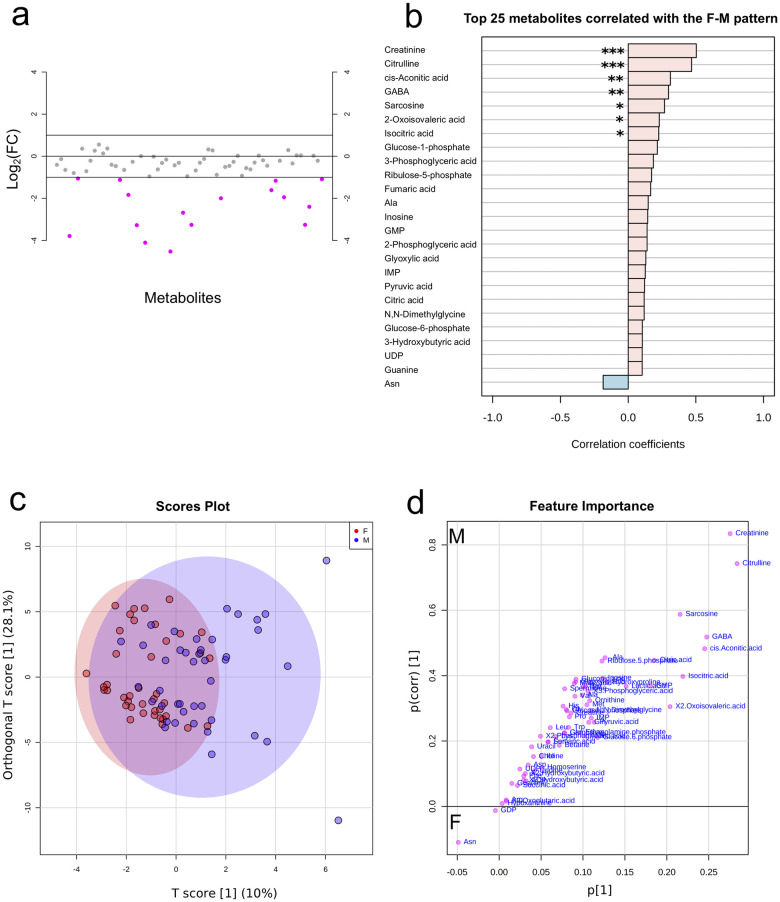
Metabolite profiles by sex. a) Metabolites identified by fold change (FC) analysis of female/male ratio with threshold of 2-FC. Values are on a log scale to show both up-regulated and down-regulated metabolites symmetrically. The plot shows metabolites that are up-regulated (positive-log scale) or down-regulated (negative log-scale). Pink symbols represent metabolites above the 2-FC threshold. b) Pattern correlation analysis showing metabolites (based on p-values) with different patterns between female and male (unadjusted top 7 metabolites significant; ***, p <0.001; **, p <0.01; *, p <0.05). Creatinine (FDR <0.001) and citrulline (FDR <0.001) remained significant after FDR correction. c) Score plot and d) Coefficient S-plot based on OPLSDA model of metabolite distribution according to sex (Y variable). Metabolites that significantly influenced the model (absolute p(corr) >0.5 and VIP ≥1.5) were creatinine, citrulline, sarcosine and GABA. For the S-plot, the y-axis represents the correlation or reliability coefficient and the x–axis represents the covariance or contributions of each metabolite to the model with respect to sex. GABA, gamma-aminobutyric acid; Ala, alanine; GMP, guanosine monophosphate; IMP, inosine monophosphate; UDP, uridine diphosphate; Asn, asparagine; F, female (n = 41); M, male (n = 42).

Initial MANOVA models were built using baseline metabolite profiles to identify any underlying variations in the study population, pre-infection, and also to identify any potential confounders. From the baseline model, metabolites were found to vary across sex, but not age. The model was then re-run with age only, and the residuals were used to identify specific metabolites that vary with sex (S1 Table). As shown in Fig 2, univariate FC analysis showed that the concentrations of 16 metabolites were lower/down-regulated (≥2-fold) in females (Fig 2A), and pattern correlation analysis showed that metabolite concentrations tend to be higher in males (Fig 2B). Of the 16, seven metabolites showing this pattern were statistically significant; creatinine (p <0.001), citrulline (p <0.001), cis-asconitic acid (p = 0.004), gamma-aminobutyric acid (GABA; p = 0.006), sarcosine (p = 0.014), 2-oxoisovaleric acid (p = 0.040), and isocitric acid (p = 0.040). Creatinine (FDR <0.001) and citrulline (FDR <0.001) remained significant after an FDR correction. Output details from the FC and pattern correlation analysis are given in S2 and S3 Tables respectively.

Based on the univariate analysis, multivariate analysis was used to determine significant metabolites associated with sex. Initial PCA analysis with a model of five components explaining 64.2% of the variability was aimed at identifying clustering according to sex. The PCA model however did not show any clear clustering by sex (see S1 Fig). This reflects the heterogeneity of data associated with human studies [67], in contrast to animal models where in-bred animals may contribute to clustering within the first few components [34]. To unmask changes, a supervised OPLSDA model was used to identify significant metabolites associated with sex (N = 83, 1 predictive and 1 orthogonal component; Q^2^ = 0.146, p = 0.001 and R^2^Y = 0.352, p = 0.033; see S2 Fig), from which a coefficient S-plot was used to identify significantly contributing metabolites discriminating between male and female. Based on the set selection criteria, males were observed to have increased concentrations of creatinine (p(corr) = 0.8, VIP = 2.9), citrulline (p(corr) = 0.7, VIP = 3.1), sarcosine (p(corr) = 0.6, VIP = 1.7), and GABA (p(corr) = 0.5, VIP = 2.4) compared to females [Fig 2C and 2D; see details in S4 Table].

### Metabolic profiles during early schistosome infection

**Fig 3:**
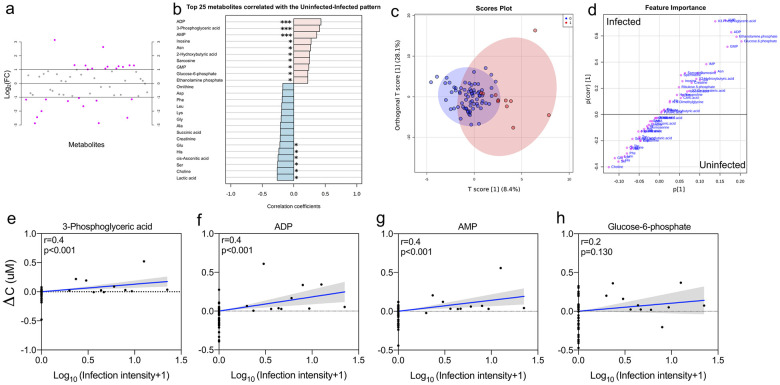
Metabolite profiles by schistosome infection status and intensity. a) Metabolites identified by fold change (FC) analysis by uninfected/infected ratio with threshold of 2-FC. Values are on a log scale to show both up-regulated and down-regulated metabolites symmetrically. The plot shows metabolites that are up-regulated (positive-log scale) or down-regulated (negative log-scale). Pink symbols represent metabolites above the 2-FC threshold. b) Pattern correlation analysis showing the top 25 metabolites (based on p-values), showing increasing and decreasing patterns with schistosome infection status (unadjusted top 16 significant; ***, p <0.001; **, p <0.01; *, p <0.05). ADP (FDR = 0.003), 3-phosphoglyceric acid (FDR = 0.003), and AMP (FDR = 0.014) remained significant after FDR correction. c) Score plot and d) coefficient S-plot based on OPLSDA model of metabolite distribution according to infection status (Y variable). Metabolites that significantly influenced the model (absolute p(corr) >0.5 and VIP ≥1.5) were AMP, 3-PG, ADP, and G6P. For the S-plot, the y-axis represents the correlation or reliability coefficient and the x–axis represents the covariance or contributions of each metabolite to the model with respect to infection status. e–h) Scatter plots showing linear regression analysis of infection intensity and change in metabolite concentration (ΔC) of the specific metabolites identified through OPLSDA as associated with schistosome infection status. Infection status was coded as 0 and 1 for uninfected (n = 72) and infected (n = 11) respectively, for n = 83 independent samples. *S*. *haematobium* infection intensity was log-transformed [log_10_ (egg count+1)]. Shaded areas indicate the 95% confidence intervals. ADP, adenosine diphosphate; AMP, adenosine monophosphate; Asn, asparagine; GMP, guanosine monophosphate; Asp, aspartic acid, Phe, phenylalanine; Leu, leucine; Lys, lysine; Gly, glycine; Ala, alanine; Glu, glutamic acid; His, histidine; Ser, serine, 3-PG, 3-phosphoglyceric acid; G6P, glucose-6-phosphate.

Due to the baseline variations in metabolite profiles related to sex, subsequent models to determine the change in metabolite profiles with schistosome infection were verified to account for potential bias. MANOVA models were built on change in metabolite profiles (ΔC) to determine any associations with *S*. *haematobium* infection, while accounting for age and sex. We found that *S*. *haematobium* status was associated with change in metabolite profiles (ΔC). For further downstream analysis, the model was re-run with age and sex only, and the residuals were used to identify metabolite features that vary by *S*. *haematobium* infection status (S5 Table). FC analysis showed that 25 metabolites were either up-regulated or down-regulated in schistosome infection more than 2-fold (Fig 3A), and metabolites showed either an increasing or decreasing pattern with schistosome infection status (Fig 3B). Of the 25, 16 metabolites showing this pattern were statistically significant. Metabolites that showed a pattern of increasing concentration with schistosome infection included adenosine diphosphate (ADP; p <0.001), 3-phosphoglyceric acid (3-PG; p <0.001), adenosine monophosphate (AMP; p <0.001), inosine (p = 0.012), asparagine (p = 0.016), 2-hydroxybutyric acid (p = 0.021), sarcosine (p = 0.023), guanosine monophosphate (GMP; p = 0.032); glucose-6-phosphate (G6P; p = 0.043), and ethanolamine phosphate (p = 0.043). Metabolites that showed a pattern of decreasing concentration with schistosome infection included lactic acid (p = 0.017), choline (p = 0.017), serine (p = 0.020), cis-asconitic acid (p = 0.023), histidine (p = 0.031), and glutamic acid (p = 0.045). ADP (FDR = 0.003), 3-PG (FDR = 0.003), and AMP (FDR = 0.014) remained significant after FDR correction. Output details from the FC and pattern correlation analysis are given in S6 and S7 Tables respectively.

Multivariate analysis was used to determine significant metabolites associated with schistosome infection. Initial PCA analysis with a model of five components explaining 57.5% of the variability, was used to identify clustering according to infection status. Likewise, the PCA model did not show any clear clustering by infection status (see S3 Fig), and heterogeneity in the data set may conceal metabolic changes characteristic of infection within the first few components [67]. To unmask such changes, a supervised OPLSDA model was used (N = 83, 1 predictive and 1 orthogonal component; Q^2^ = 0.197, p = 0.001 and R^2^Y = 0.465, p = 0.001; S4 Fig), from which a coefficient S-plot was used to identify significant metabolites discriminating between infected and uninfected children. Based on the selection criteria, *S*. *haematobium-*infected children were found to have increased concentrations of AMP (p(corr) = 0.7, VIP = 1.6), 3-PG (p(corr) = 0.7, VIP = 1.5), ADP (p(corr) = 0.6, VIP = 2.3), and G6P (p(corr) = 0.6, VIP = 1.5) [Fig 3C and 3D; see details in S8 Table].

Of interest, a MANOVA model to determine the influence of infection intensity on the significant metabolites identified by OPLSDA (accounted for age and sex) was significant (F-value = 5.178, p = 0.001; S9 Table). As shown in Fig 3E–3H, concentrations of all observed metabolites associated with schistosome infection status, increased as infection intensity increased. This relationship was significant for all metabolites except for G6P.

### Metabolite changes return to pre-infection levels following treatment

**Fig 4:**
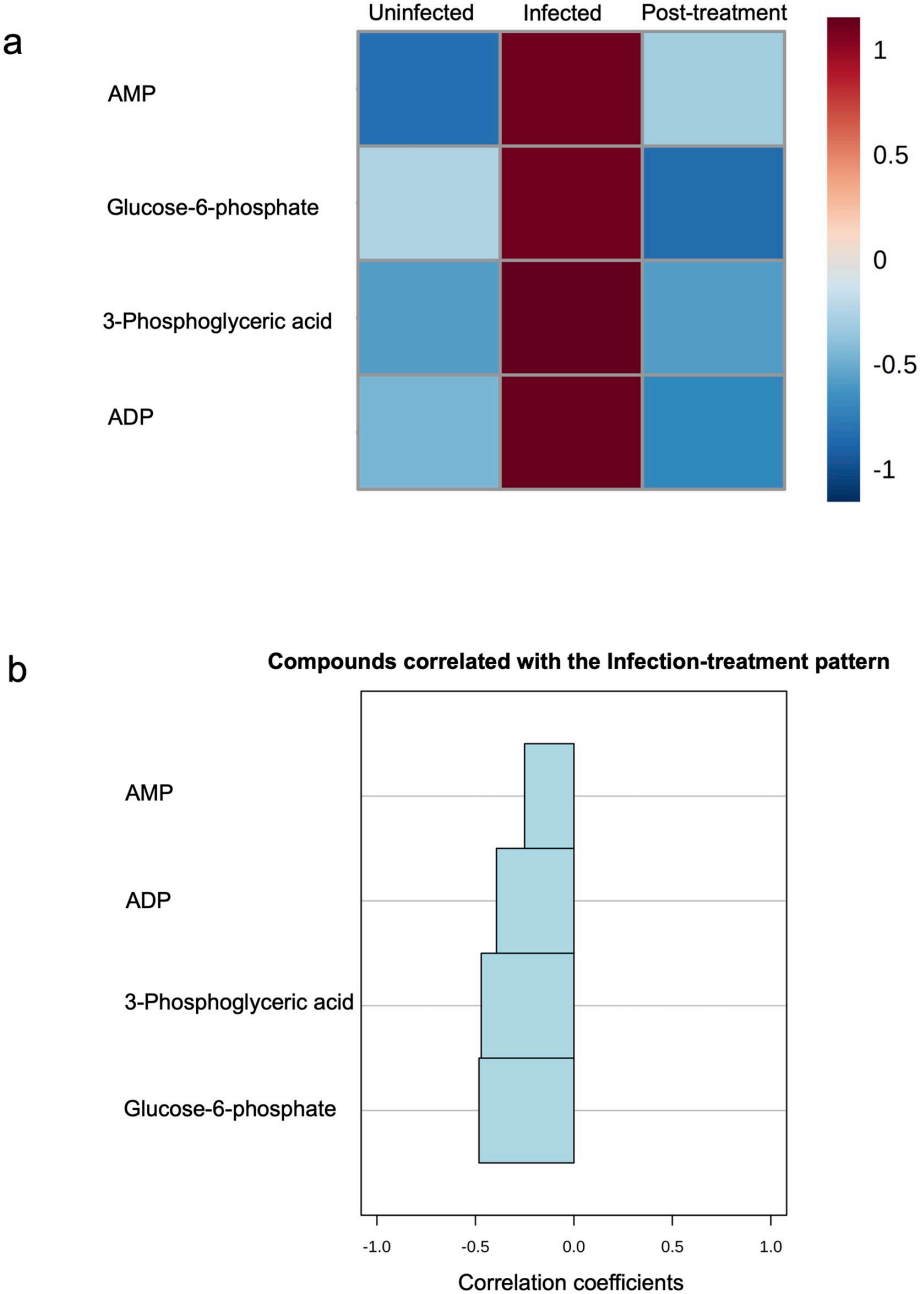
Metabolite concentrations decrease to near-uninfected levels after treatment of infection. a) Heatmap showing the mean concentration patterns of specific metabolites (associated with *S*. *haematobium* infection status) at pre-infection, infection and at post-treatment. Colour scale (1 to -1) shows Pearson’s correlation co-efficient for up-regulated (positive scale) and down-regulated metabolites (negative scale). b) Pattern correlation analysis showing decreasing metabolite concentration patterns from schistosome positive to negative post-treatment (in increasing order of absolute correlation co-efficient). ADP, adenosine diphosphate; AMP, adenosine monophosphate.

Of the 11 *S*. *haematobium*-positive children, a post-treatment follow-up sampling was conducted three months later to determine treatment efficacy, with a follow up rate of 6/11 (54.5%). Cure rate and egg reduction rates (by egg counts) were 100% in the six children followed up, and the impact of treatment on the concentration of the observed metabolites was determined in these six children. As shown in the heat map in Fig 4A, pattern analysis across all three time points showed that metabolite concentrations increased at infection and reduced to pre-infection levels, post-treatment. Although not significant (p >0.05), pattern correlation analysis (Fig 4B) showed that metabolite features exhibit a decreasing trend from infection to when children are schistosome negative (by egg count) following treatment [see details in S10 Table).

### Pathway effects of metabolite alterations associated with schistosome infection

**Fig 5:**
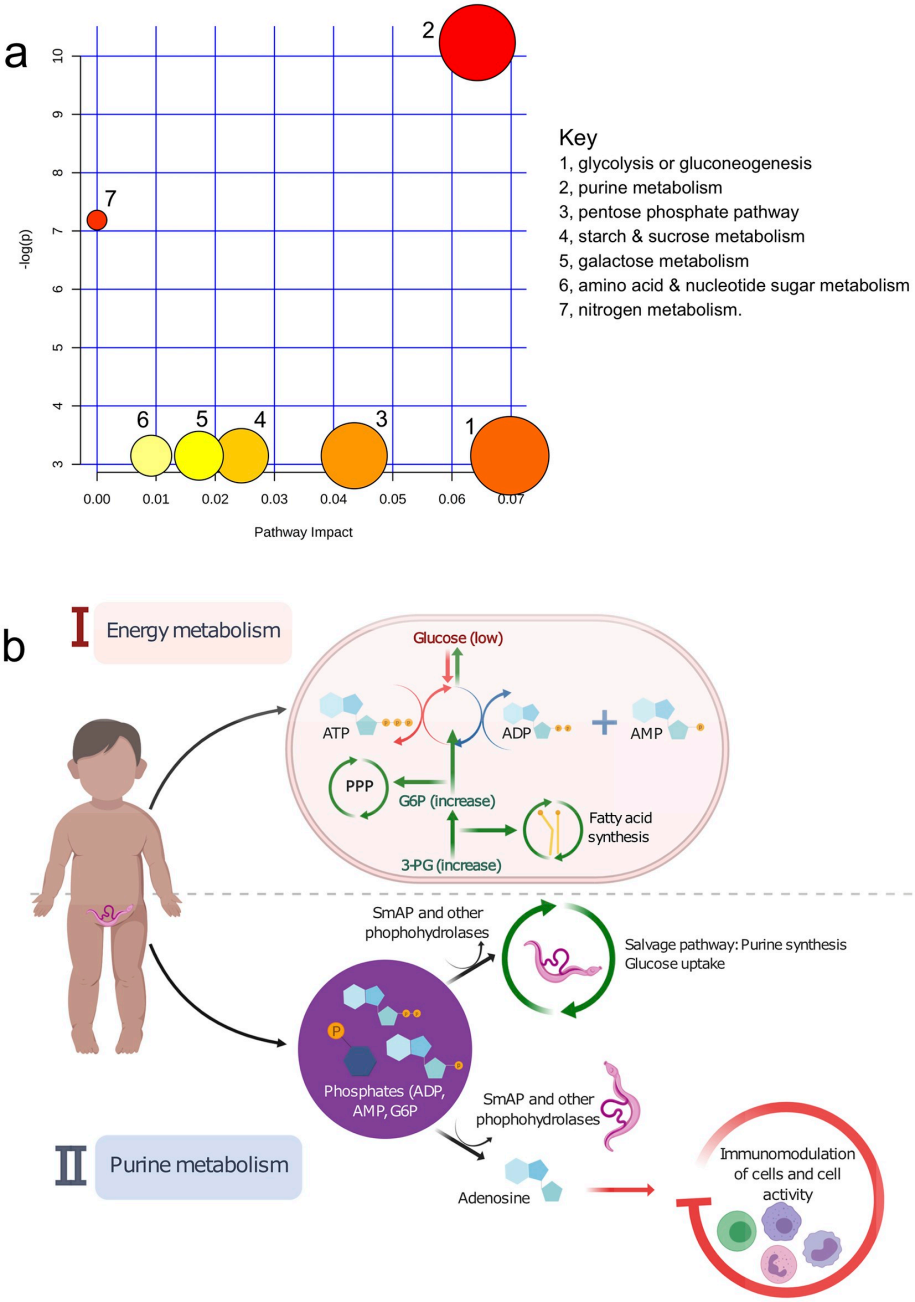
Summary of pathway analysis and biological interpretation for metabolic alterations in early *S*. *haematobium* infection. a) Pathway map showing the affected metabolic pathways. The map was generated in MetaboAnalyst and shows all matched pathways according to p-values from the pathway enrichment analysis, and pathway impact values from the pathway topology analysis. The size of each circle represents the strength of the impact on the pathway, and significance levels based on p-values range from yellow (least significant) to red (most significant). b) Pathway-based model and interpretation for the affected metabolites and corresponding metabolic pathways during schistosome infection. In response to schistosome infection and increased demands from the host, there is ↑ glycolysis, ↑ pentose phosphate pathway (PPP), ↑ fatty acid synthesis ↑ gluconeogenesis ↑glycogenolysis, as well as ↑ purine synthesis for salvage by the parasite, leading to the observed increases in the host metabolites identified (i.e. AMP, ADP, 3-PG, and G6P). AMP, adenosine monophosphate; ADP, adenosine diphosphate; ATP, adenosine triphosphate; 3-PG, 3-phosphoglyceric acid; PPP, pentose phosphate pathway; SmAP, schistosome tegumental alkaline phosphatase.

The metabolite pathway analysis assigned metabolite compounds in a total of seven pathways, which were identified together as important for the host response to schistosome infection. As shown in Fig 5A, the predominant hits were energy and purine pathways involved in glycolysis or gluconeogenesis, purine metabolism, pentose phosphate pathway (PPP), starch and sucrose metabolism, galactose metabolism, amino acid and nucleotide sugars, and nitrogen metabolism, in order of decreasing impact and statistical significance [details in S11 Table; the statistical p-values from enrichment analysis are further adjusted for multiple pathway testing). Detailed illustrations of the individual metabolism pathways from the analysis are shown in S5 Fig.

Based on the pathway analysis, we proposed a biological interpretation for the metabolite alterations observed (Fig 5B). The adult worm uses large amounts of host glucose and energy [18]. This stimulates host glycolysis and leads to an increase in host ADP and AMP [72,73]. Energy demand for increased protein synthesis could also lead to direct dephosphorylation of adenosine triphosphate (ATP), increasing host AMP. Energy demands from the parasite also increases host glycogenolysis, gluconeogenesis and fatty acid synthesis, increasing the levels of host G6P and 3-PG, essential to enhance the pentose phosphate pathway (PPP) and fatty acid synthesis respectively. In addition schistosome tegumental phosphatases and phosphohydrolases e.g. SmAP [74,75], dephosphorylate the increased exogenous host phosphate molecules (G6P, AMP, ADP), for parasite purine (i.e. adenosine) and glucose uptake. The resulting extracellular adenosine is also known to dampen host immunity [76–78] and induce host immunomodulation [22], both essential for parasite survival.

## Discussion

Host-parasite interactions are underpinned by exchange of essential metabolites between the host and the parasite [31–33]. Studies on the host and parasite metabolomes are thus informative on the nature and dynamics of these interactions. While in experimental studies it is possible to infect animals at specific time points, follow the course of infection, and investigate its impact on the host, this is not possible in human hosts. To overcome this challenge, we followed *S*. *haematobium* infection of populations living in schistosome-endemic areas in Zimbabwe, using a natural infection time-course design. We followed a cohort of Zimbabwean preschool-aged children (≤5 years old) who had never been infected by schistosomes (as confirmed by parasitological diagnosis, guardian interviews and examination of clinical records), to their first schistosome infection and curative treatment. Our study design was informed by knowledge of the exposure patterns to infective water and infection transmission dynamics in the area [79,80] and from previous sero-epidemiology studies in this age group [81,82]. Using a comprehensive mass spectrometry-based approach, we have demonstrated that first infection with the helminth *S*. *haematobium*, is associated with alterations in host metabolites, primarily linked with energy and purine metabolism. The observed changes were commensurate with increasing infection intensity, a confirmation of an association with the presence of *S*. *haematobium* infection. Metabolite levels were restored to almost pre-infection levels following curative treatment with the antiheminthic praziquantel.

We analysed the metabolic profiles of children at baseline in order to characterize metabolites in the absence of schistosome infection, as well as to account for confounding factors in subsequent analysis for any metabolite changes upon schistosome infection. Levels of creatinine, citrulline, sarcosine/*N*-methylglycine and GABA were higher in males than females. A potential explanation for this difference in amino acid metabolites could be differences in weight between the children. In adults, increased amino acid metabolites have been attributed to higher muscle mass in males [83], and this is consistent with the higher weight and associated weight–for-age Z-scores (a standardised assessment of weight in young children, relative to age [84]) in the male children included in this study. There are limited studies on protein and amino acid metabolism in healthy children, and there is a need for further studies to determine if the observations in adults translate to body profiles in young children [85].

We showed that within three months of first schistosome infection in young children, there were significant increases in AMP, ADP, 3-PG, and G6P, compared to uninfected children, and these increases correlated positively with infection intensity. Metabolic pathway analysis showed that the increases were related to energy (glycolysis, PPP, starch, and galactose) and purine metabolism. This is consistent with findings from studies conducted in experimental models of schistosome infection [34,36,86], showing that such host metabolic alterations from schistosome infection begin as early as three weeks post-infection [19].

The observed increases in discriminatory metabolites associated with infection has a physiological explanation. *S*. *haematobium* predominantly resides in the venous plexus of the bladder with direct access to the flow of nutrients in blood. Experimental studies have shown that the schistosome worm relies on the host’s glucose for survival [19]. Every five to six hours, schistosome parasites utilise their dry body weight’s worth of glucose from the host, marked by increased lactate and reduced glucose levels in the host blood stream [18,87,88]. Another consequence of schistosome infection is liver injury, as confirmed by histology in experimental studies [19] and in enzymatic human studies [89]. This liver injury is marked by stimulated host glycolysis manifested by reduced plasma glucose as well as glucose and glycogen stores in the liver, as early as 49 days post-infection [34]. Under such nutrient-poor conditions in the host, one of the main results of increased consumption of energy and ATP, is an increase in AMP and ADP, consistent with the findings in the current study [72,73]. AMP and ADP act as sensors for energy homeostasis, helping to activate alternative pathways such as glycogenolysis, gluconeogenesis and fatty acid synthesis to replenish energy stores. Also consistent with our observation of increased AMP is the enhanced energy demand for increased protein synthesis, especially for tRNA activation and guanosine triphosphate regeneration, which results in direct dephosphorylation of even more ATP to AMP by the host system [90]. Parallel to the increased glycolysis, the increased levels of G6P and 3-PG enhances the oxidative phase of the PPP (oxidising even more glucose to produce energy) to generate nicotinamide adenine dinucleotide phosphate (NADPH) for host anabolic reactions, including the biosynthesis of nucleic acids and fatty acids respectively [91]. Although this remains a pathway-based interpretation, our observation of altered energy metabolism pathways including glycolysis and the PPP, are in line with current understanding that such pathways of glucose utilisation are predominantly stimulated during schistosome infection [19,34,36]. Experimental evidence shows that the liver injury caused by schistosome infection [19] is marked by stimulated host glycolysis, manifested by reduced plasma glucose as well as glucose and glycogen stores in the liver [34]. Our results suggest an interplay between the host and the schistosome parasite, consistent with the schistosome-related morbidity observed in young children, including malnutrition, poor growth and poor physical and cognitive performance [14].

Schistosome worms have essential phosphatases and phosphohydrolases such as SmAP, that cleave exogenous phosphates to generate various reaction products [74,75]. In addition, schistosome worms lack *de novo* synthesis of purines and resort to salvaging molecules from the host using these tegumental phosphatases [92], through dephosphorylation and subsequent uptake of reaction products [93]. Thus, the observed increases in AMP, ADP, and G6P are important for parasite purine and energy uptake via phosphate cleavage using tegumental enzymes. The resulting exogenous molecules such as adenosine has anti-inflammatory properties known to dampen host immunity [76–78]. This would benefit schistosome parasites by creating a less inflammatory and immunologically friendly environment, key to survival of the parasite in the host. Data suggests that such essential functions of parasite phosphatases occur *in vivo* but less *in vitro*, consistent with our hypothesis of the benefits of such molecules for parasite survival in the host [74]. Another direct benefit of the enhanced PPP in generating molecules for fatty acid synthesis in the host is that, schistosomes rely on scavenging lipid precursors from the host to generate phospholipids, due to their inability to synthesise fatty acids *de novo* [94,95]. Schistosome lipids have also been demonstrated to stimulate immunomodulation in the host to enhance parasite survival [96,97].

The correlation between metabolic alterations and increasing egg burden is consistent with experimental studies of metabolic changes being linked to disease progression in schistosome infection [19,35]. In addition, curative treatment with praziquantel in schistosome-positive children showed a decrease in levels of the altered metabolites, three months post-treatment. Consistent with normalisation of affected pathway enzymes upon treatment of schistosome infection in mice models [36], this strengthens the idea that the observed changes are in response to or related to schistosome infection [19]. This observation is also consistent with the fact that curative treatment results in the reversal of early schistosome morbidity/pathology [13,48], and with suggestions that catch-up growth and development is possible in children, following curative praziquantel treatment [98]. We hypothesise that the effect of curative treatment would have been more marked, had the children been surveyed more than three months post-treatment, as it may take longer than that to return to pre-infection levels of the metabolites. The caveat however is that some children could get reinfected if they had been followed up within a longer time frame for post treatment sampling.

Despite allowing for analysis of matching pre- and post-infection samples for the early metabolic responses to the first *S*. *haematobium* infection in young children, the current study nonetheless had some limitations. Following a natural time-course of first schistosome infections meant that there was no control over the number of infected or uninfected children post-baseline, hence smaller sample sizes especially for schistosome positive individuals. Moreover, the analysis for return of metabolites to pre-infection levels post-treatment might be statistically significant, given a larger sample size for schistosome-positive individuals at three months and at follow up post-treatment. The use of the urine filtration technique for diagnosis, which is dependent on microscopic detection of *S*. *haematobium* eggs in urine is less sensitive to detecting very low intensity, pre-patent or single sex infections, thus underestimating infection prevalence. However, the current study allows comparison with other studies while parasitological egg count methods remain the predominant schistosome diagnostic in PSAC. Long-term studies relating measurable clinical manifestations of schistosome infection in children to such metabolic alterations, would give a stronger indication of the clinical implications of the schistosome-induced metabolic disturbances.

In conclusion, we show that in a cohort of Zimbabwean preschool-aged children (≤5 years old), the first infection with *S*. *haematobium* is associated with significant host energy and purine metabolic alterations. These changes correlated with infection intensity and resolved three months post-curative antihelminthic treatment with praziquantel. Our findings are consistent with findings from experimental schistosome studies, as well as observations of parasite-related morbidity, particularly malnutrition, poor growth and poor physical and cognitive performance in schistosome-infected children. Further mechanistic studies will contribute to more understanding of the association between metabolic disturbances and the aetiology of schistosome-related pathology in children, as well as inform the development of appropriate interventions in human helminth infections, such as nutraceuticals in child feeding programs.

## Supporting information

S1 Text Supplementary methods. (PDF)

S1 Fig Principal component analysis (PCA) of metabolite features by sex at baseline. a) Scores plot between the selected principal components. The explained variances are shown in brackets. b) Scree plot shows the variance explained by principal components. The green line on top shows the accumulated variance explained; the blue line underneath shows the variance explained by individual principal components.
(TIF)

S2 Fig OPLSDA model statistics for metabolite features by sex. a) Model overview of the OPLS-DA model for the provided dataset, showing the R^2^X, R^2^Y and Q^2^ coefficients for the groups (Male and Female). b) Permutation analysis, showing the observed and cross-validated R^2^Y and Q^2^ coefficients.
(TIF)

S3 Fig Principal component analysis (PCA) of metabolite features by infection status. a) Scores plot between the selected principal components. The explained variances are shown in brackets. b) Scree plot shows the variance explained by principal components. The green line on top shows the accumulated variance explained; the blue line underneath shows the variance explained by individual principal components.
(TIF)

S4 Fig OPLSDA model statistics for metabolite features by infection status. a) Model overview of the OPLS-DA model for the provided dataset, showing the R^2^X, R^2^Y and Q^2^ coefficients for the groups (schistosome negative and schistosome positive). b) Permutation analysis showing the observed and cross-validated R^2^Y and Q^2^ coefficients.
(TIF)

S5 Fig Detailed metabolic pathways of discriminatory metabolites for schistosome infection. a) Glycolysis/Gluconeogenesis (hits = glucose-6-phosphate). b) Purine metabolism (hits = AMP, ADP). c) Pentose phosphate pathway (hits = glucose-6-phosphate). d) Starch and sucrose metabolism (hits = glucose-6-phosphate). e) Galactose metabolism (hits = glucose-6-phosphate). f) Nitrogen metabolism (hits = glucose-6-phosphate). g) Amino, sugar and nucleotide metabolism (hits = glucose-6-phosphate). For compound colours within each metabolic pathway map–light blue are metabolites not in the data set used for pathway analysis and are used as background for enrichment analysis; other colours (varying from yellow to red) means the metabolites are in the data with different levels of significance. AMP, adenosine monophosphate; ADP, adenosine diphosphate.
(TIF)

S1 Table Baseline MANOVA output for influence of age and sex on metabolite profiles. (PDF)

S2 Table Important metabolites identified from fold change analysis by sex. (PDF)

S3 Table Correlation pattern analysis of metabolites showing a female to male pattern. (PDF)

S4 Table Output from OPLSDA model S-plot for sex, along with corresponding VIP values. (PDF)

S5 Table Follow-up MANOVA output for influence of infection on metabolite profiles. (PDF)

S6 Table Important metabolites identified from fold change analysis by infection status. (PDF)

S7 Table Correlation pattern analysis of compounds showing patterns from negative to positive for infection. (PDF)

S8 Table Output from OPLSDA model S-plot for infection, along with corresponding VIP values. (PDF)

S9 Table MANOVA output for influence of infection intensity on metabolite profiles. (PDF)

S10 Table Correlation pattern analysis of metabolites showing patterns from positive for infection to negative post-treatment. (PDF)

S11 Table Analysis output from metabolite pathway analysis. (PDF)
