# Supplementary material for: Schistosoma haematobium infection is associated with alterations in energy and purine-related metabolism in preschool-aged children
Source: PLoS Negl Trop Dis. 2020 Dec 14;14(12):e0008866. doi: 10.1371/journal.pntd.0008866 (PMC7735607; doi:10.1371/journal.pntd.0008866)
Supplement: S1 Text — (PDF) [file pntd.0008866.s001.pdf]

Supplementary Methods

Instrumentation

Capillary electrophoresis time-of-flight mass spectrometry (CE–TOF–MS) was carried out using an Agilent CE–TOF–MS system (Agilent Technologies Inc. Waldbronn, Germany) system, equipped with an Agilent 6210 Time of Flight mass spectrometer (TOF–MS), Agilent 1100 isocratic High-performance liquid chromatography (HPLC) pump, Agilent G1603A capillary electrophoresis mass spectrometry (CE–MS) adapter kit, and Agilent G1607A capillary electrophoresis electrospray ionization mass spectrometry (CE–ESI–MS) sprayer kit (all from Agilent Technologies, Waldbronn, Germany). System control, data acquisition and evaluation were controlled by Agilent G2201AA ChemStation software version B.03.01 for CE (Agilent Technologies). To facilitate thermostating of the capillary, the CE–MS adapter kit includes a capillary cassette. The CE−ESI–MS sprayer kit simplifies coupling the CE system with MS systems equipped with an electrospray source. The sprayer is designed to give an orthogonal flow in order to reduce the detrimental effects caused by the charged particles or droplets [1].

Metabolite measurement

Compounds were measured in the cation and anion modes of CE–TOF–MS-based metabolome analysis [2-4]. To improve the quality of the CE–MS analysis, samples were diluted 1:2 and 1:5 for cation and anion modes respectively.

Cationic metabolites (i.e. cation mode) were analysed with a fused silica capillary (i.d. 50 μm × 80 cm), using Cation Buffer Solution (p/n: H3301-1001; HMT) as both the run and rinse buffer. Sample injection was done at a pressure of 50 mbar for 10 seconds at an applied CE voltage of 27 kV. Electrospray ionization mass spectrometry (ESI–MS) was conducted in the positive ion mode with a capillary voltage of 4,000 V. Mass spectrometer (MS) scanning range

was *m/z* 50–1,000 using an HMT in-house sheath liquid (p/n: H3301-1020). All other conditions were as used in cation analysis mass spectrometry [2]. Anionic metabolites (i.e. anion mode) were analysed with a fused silica capillary (i.d. 50 µm × 80 cm), using Anion Buffer Solution (p/n: H3302-1021; HMT) as both the run and rinse buffer. Sample injection was done at a pressure of 50 mbar for 25 seconds at an applied CE voltage of 30 kV. ESI-MS was conducted in the negative ion mode with a capillary voltage of 3,500 V. MS scanning range was *m/z* 50–1,000 using an HMT in-house sheath liquid (p/n : H3301-1020). All other conditions were as used in anion analysis mass spectrometry [4].

Statistical analysis

Data analyses were performed using SPSS version 22 (IBM Corp.), GraphPad Prism version 8.2.0 (GraphPad Software, Inc), and MetaboAnalyst 4.0. MetaboAnalyst is a comprehensive, Web-based tool that supports analysis of metabolomic data using a range of univariate, multivariate, and machine-learning methods and tools [5-7].

Data pre-processing. Prior to any downstream analysis, a data integrity check was performed using MetaboAnalyst. First, the entire data set from all three time points was checked for missing values. Overall, metabolite concentration values should be non-negative and without missing values (in this case "N.D"); these cause difficulties in data normalisation and downstream analysis. Since missing or zero values were caused by metabolites with abundance below the detection limit, and not a mere absence, these were replaced by a small pseudo value (i.e. half the minimum positive value in the entire data set= 0.15 µM).

Metabolite data were then processed to remove data for metabolites with a constant value across all samples. By default, MetaboAnalyst removes data for metabolites with a constant or a single value across samples. For example, a metabolite with concentration values of 0.15 µM across all 83 samples will be removed. For the metabolite data set at baseline, six metabolites

were found and removed (Adenosine triphosphate (ATP), Anthranilic acid, Betaine aldehyde + H2O, Dihydroxyacetone phosphate, Glycerol-3-phosphate, and Phosphoenolpyruvic acid).

For change in metabolite concentrations in response to schistosome infection, the change in concentration of metabolites between the two time points at baseline (C1) and at follow up for infection (C2) was calculated as ΔC μM = C2-C1 (μM) and used for subsequent analysis. The resulting data set was then processed to exclude data for metabolites with a constant value across all samples. Similarly, the six metabolites which were found and removed from the baseline metabolite data set were also removed by default. To improve statistical power, eight additional metabolites with less than n=10 non-zero ΔC μM values across all samples were excluded from analysis with the change in concentration metabolite data set (14 metabolites in total; 2-Phosphoglyceric acid, Anthranilic acid, ATP, Betaine aldehyde +H2O, Dihydroxyacetone phosphate, Fumaric acid, GDP, Glucose 1-phosphate, Glycerol 3-phosphate, Glyoxylic acid, Guanine, Phosphoenolpyruvic acid, UDP, and Uracil).

For all analyses, data were processed by range scaling (mean-centred and divided by the value range of each variable); this allows for biologically-related scaling and ensures that all metabolites are treated as equally important [8].

Multivariate analysis of variance (MANOVA) using SPSS. To determine if the mean differences between groups in the sample metadata on the metabolite data set were likely due to chance, a Multivariate analysis of variance (MANOVA; SPSS) with sequential sums of squares was used, as recommended for pathogen related studies [9]. In this case, the effects of each term are adjusted only for the effects of terms preceding it in the model; the variables of interest are thus entered other confounding variable [9]. Where a variable was found to be significant, the model was re-run excluding the significant variable, to account for the effects of all other variables. This was to ensure that the confounding effects of factors such as age

and sex were already accounted for, prior to downstream analysis to determine to determine the most relevant metabolites accounting for differences in metabolite profiles between groups of interest. The residuals from the resulting model were saved and subjected to further univariate and multivariate analysis to identify significant metabolites (MetaboAnalyst).

Age and sex-dependent effects exist for metabolites [10, 11] and for schistosome infection [12, 13]. A MONOVA model for age (years), sex, and their interaction (in that order) was used to determine any underlying age and sex-related associations at baseline, which may account for difference in metabolite concentrations post-infection; sex remained significant. To further identify specific metabolites associated with sex at baseline (significant in the initial model), the MANOVA model was re-run with age and the residuals saved for further analysis in MetaboAnalyst. To determine if the change in metabolite concentrations due to schistosome infection are is likely due by chance, a MONOVA model for age (years), sex, infection status and all interactions was run (in that order); infection status remained significant. To further identify metabolites associated with schistosome infection at follow-up (significant in the initial model), the MANOVA model was re-run with age, sex, and their interactions, and residuals saved for further analysis.

Fold change and correlation analysis. Univariate analysis was first used to obtain an overview about features that are potentially significant in discriminating the conditions under study (MetaboAnalyst). A fold change (FC) analysis was first done to compare absolute value changes in metabolites between two group means. A concentration ratio (i.e. between the two groups) of at least a 2-fold was considered significant [14]. A Pearson's pattern correlation analysis with FDR correction (<0.05) [15] was also used to determine linear/periodic trends, and to show metabolite variation patterns under different conditions.

PCA and OPLSDA. More stringent multivariate analysis was then used to identify significant metabolites associated with sex and schistosome infection. For an informative first-hand look at the data set, an unsupervised Principal Component Analysis (PCA) was employed to assess clustering trends and group separation in the data set. To test the hypothesis that specific metabolite signatures are associated with schistosome infection, a supervised multiple regression analysis method, Orthogonal Projections to Latent Structures Discriminant Analysis (OPLSDA) was used to discriminate groups and identify the differentially expressed metabolites that drive group separation [16]. OPLSDA is modified version of the Partial Least Squares - Discriminant Analysis (PLSDA) [17], and has the capability to distinguish between variations in a data set both relevant and irrelevant to predicting groups, while incorporating an Orthogonal Signal Correction (OSC) filtering [18] into a Partial Least Squares (PLS) model. The model relates numerous response variables (Y), in this case the metabolite data set, and X blocks of matrices (in this case, using dummy variables 0/1 for groups) by a linear multivariate model. It then separates the systematic variation between groups into two predictive (covariance between X and Y; between group variation) and orthogonal (systematic variation in X that is unrelated to Y; within group variation) components, free of interfering structured variation. Model statistics, R^2 and Q^2, were calculated for each model and used to assess the degree of fit and predictive reliability of the OPLSDA model respectively [19]. R^2 represents the fraction of the variance explained by a component in the model and is expressed as R^2= (1−RSS/SS), where RSS/SS is the fitted residual sum of squares or the sum squares of the response variables respectively. The Q^2 is the cross-validated R^2, expressed as (1-PRESS/SS), where PRESS is prediction error of the sum of squares and SS is the sum squares of the response variables [19].

A permutation testing that assumes there is no difference between any two groups compared was used to cross-validate and ensure the model was reliable and not over-fitted [17]. In

summary, sample groups were randomly permuted, and a new classification model calculated. Model performance was then assessed by the Q^2 and R^2 diagnostic statistics, expected to be lower than that obtained for the original unpermuted data set. The permutations were repeated 1000 times and the diagnostic statistics obtained were used to create a null distribution, H_0, of models expected to be non-significant. The diagnostic statistics for the OPLSDA model from the original data set was related to that of the H_0 distribution from the permuted data sets to determine the statistical significance (p-value with threshold <0.05) of the OPLSDA model:

$$p = \frac{1 + \#(Q^2p \geq Q^2)}{N}$$

Where *N* is the number of permutations, $\#(Q^2p \geq Q^2)$ is the number of elements in the null distribution H_0 which are greater or equal to the Q^2 for the original data set (or otherwise R^2).

Selection of significant metabolites. For all valid OPLSDA models, an S-plot showing the variable importance in a model, combining the covariance or contributions [X-axis; p(1)] and the correlation or reliability coefficient [Y-axis; p(corr)] loading profile was generated. This was used to identify and select significant metabolites with the highest correlation coefficient within groups and with the highest contribution to the model separation between groups. The p(corr) values are robust to variable selection in the OPLSDA model and are thus comparable between models [20]. The variable importance in the projection (VIP), a weighted sum of squares of the PLS loadings, taking into account the amount of explained group variation in each dimension, was calculated for each component. As recommended, a combination of the S-plot, using an absolute p(corr) >0.5 and a VIP value cut-off ≥1.5 were used to select significant metabolites [20, 21].

Pathway enrichment analysis and topology analysis. We used the pathway enrichment analysis (quantitative enrichment analysis using the compound concentration values). This is a sensitive method with the potential to identify subtle but consistent changes amongst

metabolites involved in the same biological pathway. As MetaboAnalyst is a web-based tool, the Global Test was used and p-values were approximated based on the asymptotic distribution without using permutations; this is suitable when most relevant pathways are to be identified, and thus the rank of the pathway is most essential. The Global Test allows the use of metabolites selected based on prior analysis, and to investigate groups of differentially expressed metabolites of biological interest [22]. The pathway topology analysis in MetaboAnalyst takes into consideration the structure of biological pathways to estimate significant pathways that change under different conditions. We used the out-degree centrality, which represents the number of links that are initiated from a node (metabolite); it is assumed that nodes upstream will have regulatory roles for the downstream nodes, not vice versa (i.e. assuming that upstream metabolites will have regulatory effects on downstream metabolites but not vice versa, and that changes in more important positions of a network will trigger a more severe impact) [23].

References

1. Voyksner RD, Lee H. Improvements in LC/electrospray ion trap mass spectrometry performance using an off-axis nebulizer. Analytical Chemistry. 1999;71(7):1441-7. doi: DOI 10.1021/ac980995s. PubMed PMID: WOS:000079593900042.
2. Soga T, Heiger DN. Amino acid analysis by capillary electrophoresis electrospray ionization mass spectrometry. Anal Chem. 2000;72(6):1236-41. doi: 10.1021/ac990976y. PubMed PMID: 10740865.
3. Soga T, Ohashi Y, Ueno Y, Naraoka H, Tomita M, Nishioka T. Quantitative metabolome analysis using capillary electrophoresis mass spectrometry. J Proteome Res. 2003;2(5):488-94. doi: 10.1021/pr034020m. PubMed PMID: WOS:000186002600004.
4. Soga T, Ueno Y, Naraoka H, Ohashi Y, Tomita M, Nishioka T. Simultaneous determination of anionic intermediates for Bacillus subtilis metabolic pathways by capillary electrophoresis electrospray ionization mass spectrometry. Anal Chem. 2002;74(10):2233-9. doi: 10.1021/ac020064n. PubMed PMID: 12038746.
5. Chong J, Soufan O, Li C, Caraus I, Li S, Bourque G, et al. MetaboAnalyst 4.0: towards more transparent and integrative metabolomics analysis. Nucleic Acids Res. 2018;46(W1):W486-W94. doi: 10.1093/nar/gky310. PubMed PMID: 29762782; PubMed Central PMCID: PMCPMC6030889.
6. Xia J, Psychogios N, Young N, Wishart DS. MetaboAnalyst: a web server for metabolomic data analysis and interpretation. Nucleic Acids Res. 2009;37(Web Server

issue):W652-60. doi: 10.1093/nar/gkp356. PubMed PMID: 19429898; PubMed Central PMCID: PMCPMC2703878.
7. Xia J, Wishart DS. Web-based inference of biological patterns, functions and pathways from metabolomic data using MetaboAnalyst. Nature Protocols. 2011;6:743. doi: 10.1038/nprot.2011.319.
8. van den Berg RA, Hoefsloot HC, Westerhuis JA, Smilde AK, van der Werf MJ. Centering, scaling, and transformations: improving the biological information content of metabolomics data. BMC Genomics. 2006;7:142. doi: 10.1186/1471-2164-7-142. PubMed PMID: 16762068; PubMed Central PMCID: PMCPMC1534033.
9. Mutapi F, Roddam A. p values for pathogens: statistical inference from infectious-disease data. Lancet Infect Dis. 2002;2(4):219-30. PubMed PMID: 11937422.
10. Gu H, Pan Z, Xi B, Hainline BE, Shanaiah N, Asiago V, et al. 1H NMR metabolomics study of age profiling in children. NMR Biomed. 2009;22(8):826-33. doi: 10.1002/nbm.1395. PubMed PMID: 19441074; PubMed Central PMCID: PMCPMC4009993.
11. Fan S, Yeon A, Shahid M, Anger JT, Eilber KS, Fiehn O, et al. Sex-associated differences in baseline urinary metabolites of healthy adults. Sci Rep. 2018;8(1):11883. doi: 10.1038/s41598-018-29592-3. PubMed PMID: 30089834; PubMed Central PMCID: PMCPMC6082868.
12. Woolhouse ME, Mutapi F, Ndhlovu PD, Chandiwana SK, Hagan P. Exposure, infection and immune responses to Schistosoma haematobium in young children. Parasitology. 2000;120 (Pt 1):37-44. doi: 10.1017/s0031182099005156. PubMed PMID: 10726264.
13. Rudge JW, Stothard JR, Basanez MG, Mgeni AF, Khamis IS, Khamis AN, et al. Micro-epidemiology of urinary schistosomiasis in Zanzibar: Local risk factors associated with distribution of infections among schoolchildren and relevance for control. Acta Trop. 2008;105(1):45-54. doi: 10.1016/j.actatropica.2007.09.006. PubMed PMID: 17996207.
14. Patterson TA, Lobenhofer EK, Fulmer-Smentek SB, Collins PJ, Chu TM, Bao W, et al. Performance comparison of one-color and two-color platforms within the MicroArray Quality Control (MAQC) project. Nat Biotechnol. 2006;24(9):1140-50. doi: 10.1038/nbt1242. PubMed PMID: 16964228.
15. Benjamini Y, Hochberg Y. Controlling the false discovery rate: a practical and powerful approach to multiple testing. Journal of the Royal statistical society: series B (Methodological). 1995;57(1):289-300.
16. Wiklund S, Johansson E, Sjostrom L, Mellerowicz EJ, Edlund U, Shockcor JP, et al. Visualization of GC/TOF-MS-based metabolomics data for identification of biochemically interesting compounds using OPLS class models. Analytical Chemistry. 2008;80(1):115-22. doi: 10.1021/ac0713510. PubMed PMID: WOS:000252026900021.
17. Szymanska E, Saccenti E, Smilde AK, Westerhuis JA. Double-check: validation of diagnostic statistics for PLS-DA models in metabolomics studies. Metabolomics. 2012;8(Suppl 1):3-16. doi: 10.1007/s11306-011-0330-3. PubMed PMID: 22593721; PubMed Central PMCID: PMCPMC3337399.
18. Wold S, Antti H, Lindgren F, Ohman J. Orthogonal signal correction of near-infrared spectra. Chemometr Intell Lab. 1998;44(1-2):175-85. doi: Doi 10.1016/S0169-7439(98)00109-9. PubMed PMID: WOS:000077949300013.
19. Wold S, Sjostrom M, Eriksson L. PLS-regression: a basic tool of chemometrics. Chemometr Intell Lab. 2001;58(2):109-30. doi: Doi 10.1016/S0169-7439(01)00155-1. PubMed PMID: WOS:000172360800006.
20. Wheelock AM, Wheelock CE. Trials and tribulations of 'omics data analysis: assessing quality of SIMCA-based multivariate models using examples from pulmonary

medicine. Mol Biosyst. 2013;9(11):2589-96. doi: 10.1039/c3mb70194h. PubMed PMID: 23999822.
21. Du Z, Shu Z, Lei W, Li C, Zeng K, Guo X, et al. Integration of Metabonomics and Transcriptomics Reveals the Therapeutic Effects and Mechanisms of Baoyuan Decoction for Myocardial Ischemia. Front Pharmacol. 2018;9:514. doi: 10.3389/fphar.2018.00514. PubMed PMID: 29875658; PubMed Central PMCID: PMCPMC5974172.
22. Goeman JJ, van de Geer SA, de Kort F, van Houwelingen HC. A global test for groups of genes: testing association with a clinical outcome. Bioinformatics. 2004;20(1):93-9. Epub 2003/12/25. doi: 10.1093/bioinformatics/btg382. PubMed PMID: 14693814.
23. Aittokallio T, Schwikowski B. Graph-based methods for analysing networks in cell biology. Brief Bioinform. 2006;7(3):243-55. doi: 10.1093/bib/bbl022. PubMed PMID: 16880171.
