# Supplementary figures and images for: Schistosoma haematobium infection is associated with alterations in energy and purine-related metabolism in preschool-aged children
Source: PLoS Negl Trop Dis. 2020 Dec 14;14(12):e0008866. doi: 10.1371/journal.pntd.0008866 (PMC7735607; doi:10.1371/journal.pntd.0008866)

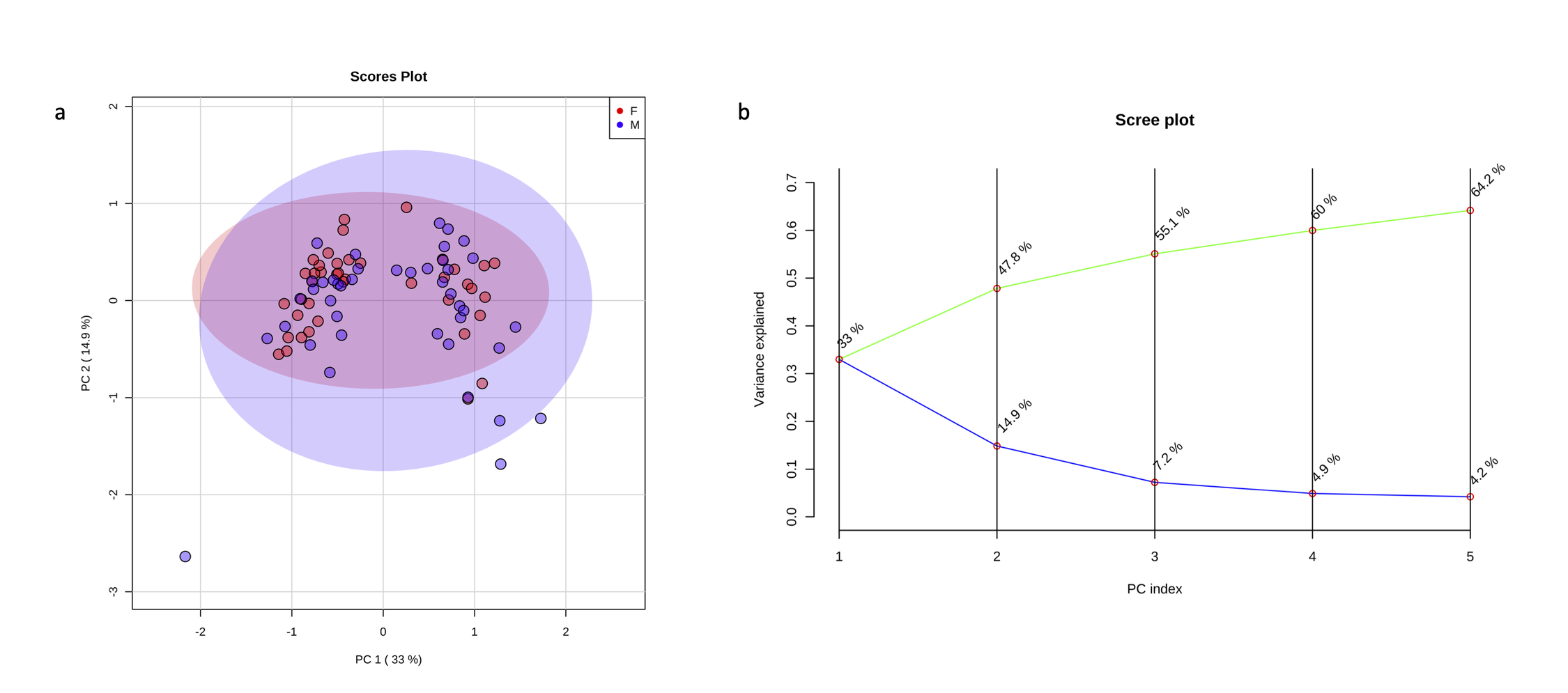

Supplement: S1 Fig — a) Scores plot between the selected principal components. The explained variances are shown in brackets. b) Scree plot shows the variance explained by principal components. The green line on top shows the accumulated variance explained; the blue line underneath shows the variance explained by individual principal components. (TIF) [file pntd.0008866.s002.tif]

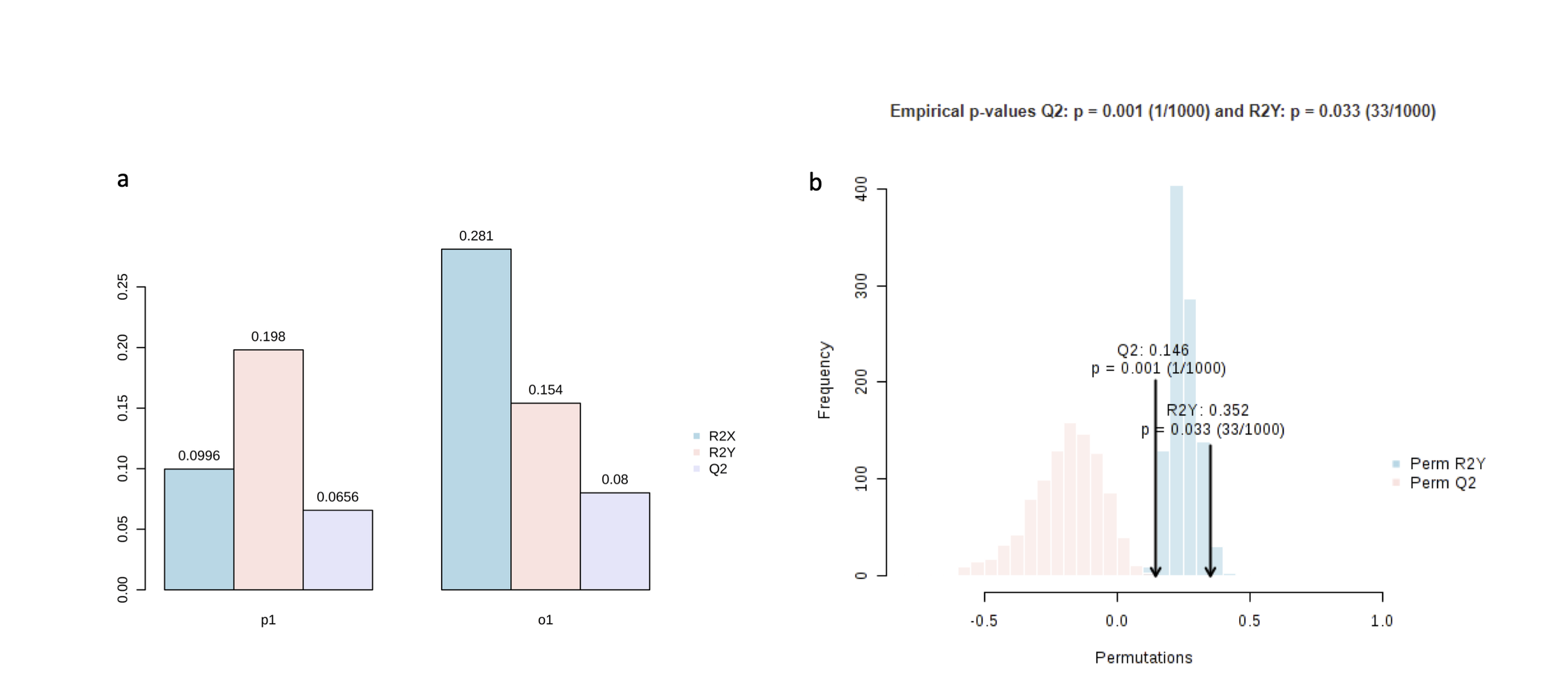

Supplement: S2 Fig — a) Model overview of the OPLS-DA model for the provided dataset, showing the R2X, R2Y and Q2 coefficients for the groups (Male and Female). b) Permutation analysis, showing the observed and cross-validated R2Y and Q2 coefficients. (TIF) [file pntd.0008866.s003.tif]

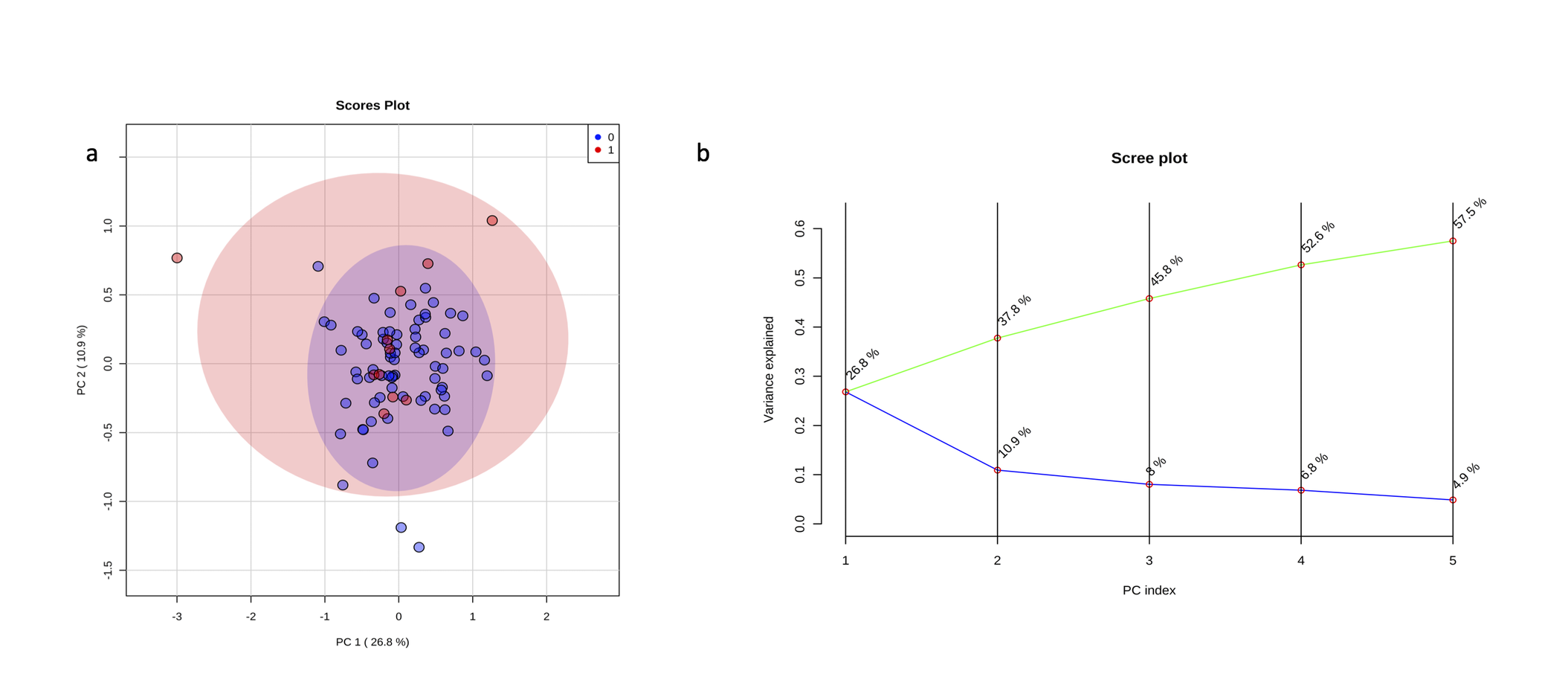

Supplement: S3 Fig — a) Scores plot between the selected principal components. The explained variances are shown in brackets. b) Scree plot shows the variance explained by principal components. The green line on top shows the accumulated variance explained; the blue line underneath shows the variance explained by individual principal components. (TIF) [file pntd.0008866.s004.tif]

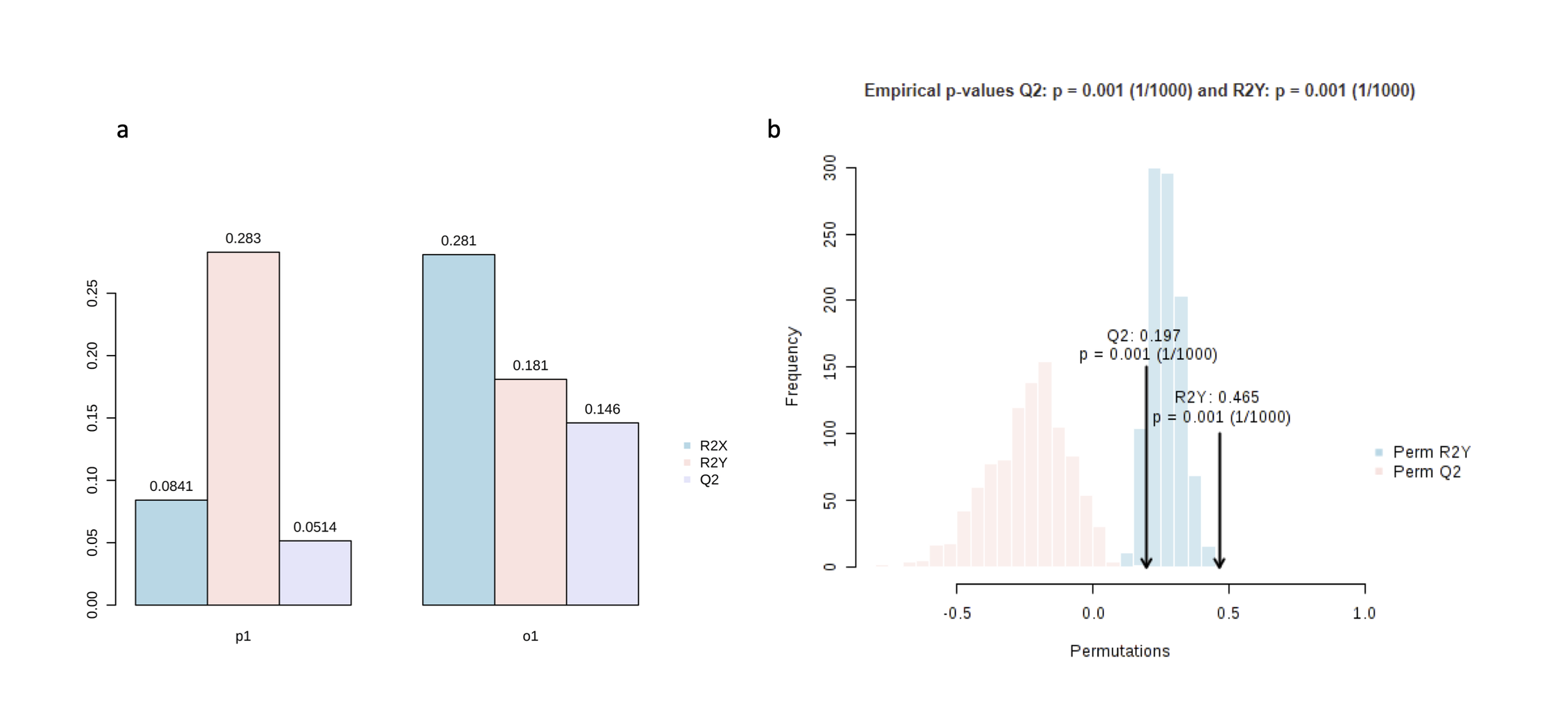

Supplement: S4 Fig — a) Model overview of the OPLS-DA model for the provided dataset, showing the R2X, R2Y and Q2 coefficients for the groups (schistosome negative and schistosome positive). b) Permutation analysis showing the observed and cross-validated R2Y and Q2 coefficients. (TIF) [file pntd.0008866.s005.tif]

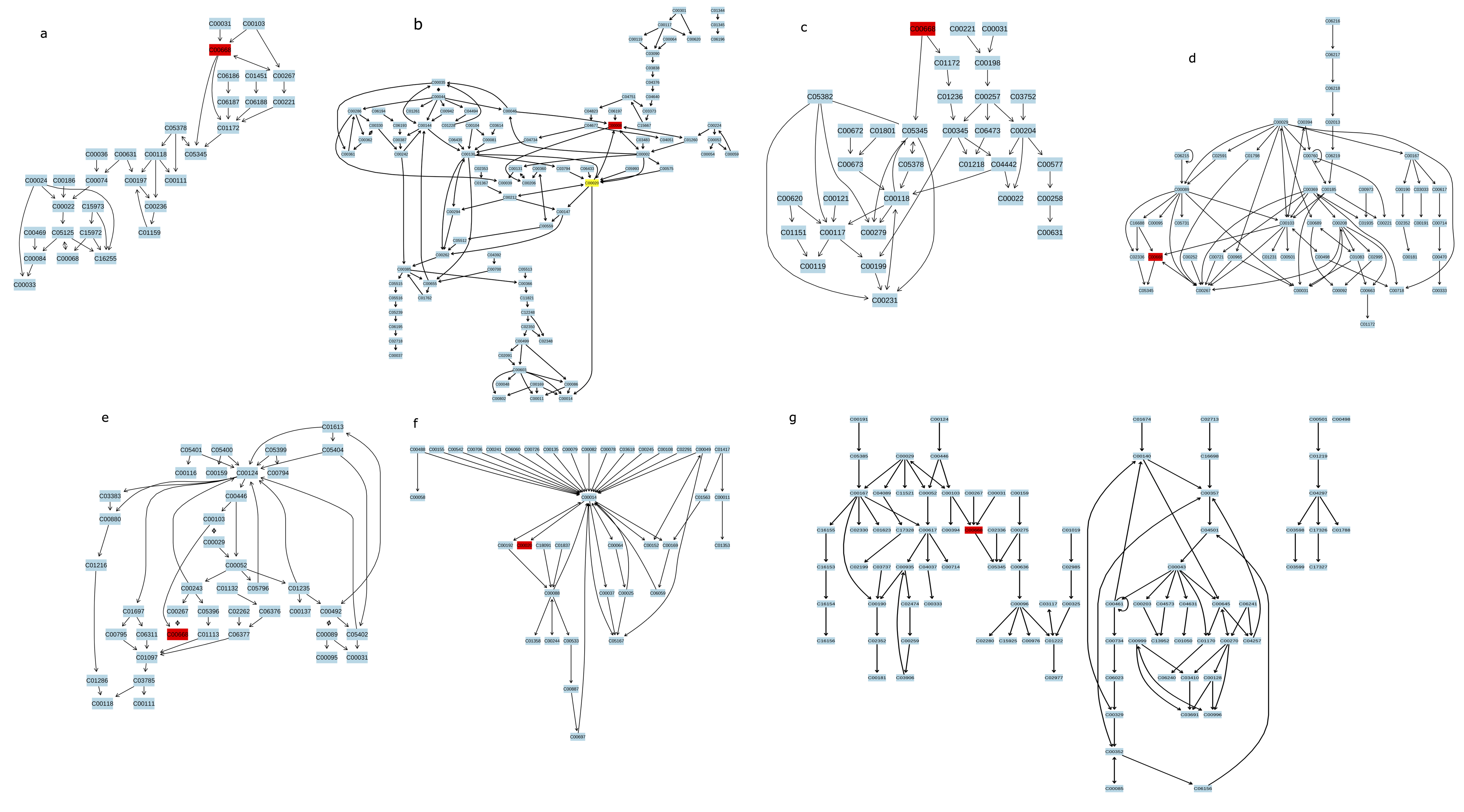

Supplement: S5 Fig — a) Glycolysis/Gluconeogenesis (hits = glucose-6-phosphate). b) Purine metabolism (hits = AMP, ADP). c) Pentose phosphate pathway (hits = glucose-6-phosphate). d) Starch and sucrose metabolism (hits = glucose-6-phosphate). e) Galactose metabolism (hits = glucose-6-phosphate). f) Nitrogen metabolism (hits = glucose-6-phosphate). g) Amino, sugar and nucleotide metabolism (hits = glucose-6-phosphate). For compound colours within each metabolic pathway map–light blue are metabolites not in the data set used for pathway analysis and are used as background for enrichment analysis; other colours (varying from yellow to red) means the metabolites are in the data with different levels of significance. AMP, adenosine monophosphate; ADP, adenosine diphosphate. (TIF) [file pntd.0008866.s006.tif]
