# Supplementary material for: Schistosoma haematobium infection is associated with alterations in energy and purine-related metabolism in preschool-aged children
Source: PLoS Negl Trop Dis. 2020 Dec 14;14(12):e0008866. doi: 10.1371/journal.pntd.0008866 (PMC7735607; doi:10.1371/journal.pntd.0008866)
Supplement: S1 Table — (PDF) [file pntd.0008866.s007.pdf]

S1 Table: Baseline MANOVA output for influence of age and sex on metabolite profiles

Variable	F value	Hypothesis/Total df	Error df	Partial Eta-squared	p-value
Intercept	0	64	16	0	1.000
Age	1.259	64	16	0.834	0.314
Sex	2.736	64	16	0.916	0.014
Age * Sex	0.619	64	16	0.712	0.910
Equation: Intercept + Age + Sex +Age * Sex					
	**Model to obtain residuals**				
Intercept	1.066	64	18	0.791	0.462
Age	1.133	64	18	0.801	0.400
Equation: Intercept + Age					

df, degrees of freedom.
