# Supplementary material for: Schistosoma haematobium infection is associated with alterations in energy and purine-related metabolism in preschool-aged children
Source: PLoS Negl Trop Dis. 2020 Dec 14;14(12):e0008866. doi: 10.1371/journal.pntd.0008866 (PMC7735607; doi:10.1371/journal.pntd.0008866)
Supplement: S2 Table — (PDF) [file pntd.0008866.s008.pdf]

S2 Table: Important metabolites identified from fold change analysis by sex

Metabolite	Fold Change (FC)	$\log_2$ (FC)
3-Phosphoglyceric acid	0.48062	-1.057
β-Alanine	0.46924	-1.0916
cis-Aconitic acid	0.45987	-1.1207
Sarcosine	0.44656	-1.1631
Ribulose-5-phosphate	0.32765	-1.6098
Citrulline	0.28031	-1.8349
Spermidine	0.25941	-1.9467
Inosine	0.25062	-1.9965
Uracil	0.18962	-2.3988
Glyoxylic acid	0.15585	-2.6817
Guanine	0.10453	-3.258
Uridine diphosphate	0.10453	-3.258
Creatinine	0.10332	-3.2748
2-Phosphoglyceric acid	0.072414	-3.7876
Fumaric acid	0.058175	-4.1035
Glucose-1-phosphate	0.043478	-4.5236

Table shows metabolites ranked in order of decreasing absolute fold change. Analysis is according to female/male ratio.
