# Supplementary material for: Schistosoma haematobium infection is associated with alterations in energy and purine-related metabolism in preschool-aged children
Source: PLoS Negl Trop Dis. 2020 Dec 14;14(12):e0008866. doi: 10.1371/journal.pntd.0008866 (PMC7735607; doi:10.1371/journal.pntd.0008866)
Supplement: S3 Table — (PDF) [file pntd.0008866.s009.pdf]

S3 Table: Correlation pattern analysis of metabolites showing a female to male pattern

Metabolite	Correlation	t-statistic	p-value	FDR
Creatinine	0.50335	5.2427	1.23E-06	7.90E-05
Citrulline	0.46873	4.7757	7.85E-06	0.00025121
cis-Aconitic acid	0.31171	2.9525	0.0041229	0.087955
gamma-aminobutyric acid (GABA)	0.2977	2.8066	0.0062697	0.10031
Sarcosine	0.26854	2.509	0.014102	0.1805
2-Oxoisovaleric acid	0.2305	2.1319	0.036047	0.36727
Isocitric acid	0.22574	2.0855	0.040171	0.36727
Glucose-1-phosphate	0.21615	1.9925	0.049688	0.3975
3-Phosphoglyceric acid	0.18689	1.7122	0.090693	0.60492
Asparagine	-0.18475	-1.6918	0.094519	0.60492
Ribulose-5-phosphate	0.17364	1.5868	0.11644	0.67749
Fumaric acid	0.16667	1.5213	0.13207	0.70437
β-Alanine	0.14805	1.3473	0.18164	0.77685
Inosine	0.14542	1.3228	0.18961	0.77685
Guanosine monophosphate (GMP)	0.14044	1.2766	0.2054	0.77685
2-Phosphoglyceric.acid	0.14004	1.2729	0.20669	0.77685
Glyoxylic acid	0.13052	1.1848	0.23956	0.77685
Inosine monophosphate (IMP)	0.1264	1.1468	0.25483	0.77685
Pyruvic acid	0.12088	1.0959	0.27636	0.77685
Citric acid	0.11845	1.0737	0.28617	0.77685
N.N-Dimethylglycine	0.11823	1.0716	0.28709	0.77685
Glucose-6-phosphate	0.10428	0.94363	0.34817	0.77685
3-Hydroxybutyric acid	0.10402	0.94127	0.34936	0.77685
Guanine	0.10342	0.93577	0.35217	0.77685
Uridine diphosphate (UDP)	0.10342	0.93577	0.35217	0.77685
Adenosine monophosphate (AMP)	0.10328	0.93453	0.35281	0.77685
Hydroxyproline	0.10234	0.92596	0.35722	0.77685
Uracil	0.095602	0.86438	0.38993	0.77685
Methionine	0.095524	0.86367	0.39032	0.77685
Arginine	-0.095024	-0.8591	0.39282	0.77685
Lysine	-0.093629	-0.84638	0.39984	0.77685
Spermidine	0.092362	0.83483	0.40627	0.77685
Glutamine	0.090823	0.8208	0.41417	0.77685
Aspartic acid	-0.090674	-0.81944	0.41494	0.77685
Phenylalanine	-0.086671	-0.78298	0.43592	0.77685
Hypoxanthine	-0.086471	-0.78117	0.43698	0.77685
Malic acid	0.079649	0.71913	0.47413	0.82012
Betaine	-0.075391	-0.68045	0.49816	0.839
Isoleucine	-0.067526	-0.60912	0.54415	0.89296
Succinic acid	-0.063331	-0.57113	0.56949	0.90425
Ethanolamine phosphate	0.060998	0.55	0.58383	0.90425
Creatine	-0.057916	-0.52212	0.60301	0.90425
Histidine	0.055047	0.49617	0.62112	0.90425
Lactic acid	0.049938	0.45001	0.65391	0.90425

Metabolite	Correlation	t-statistic	p-value	FDR
2-Oxoglutaric acid	0.048837	0.44006	0.66106	0.90425
Uridine	-0.04522	-0.4074	0.68479	0.90425
Carnosine	0.043415	0.3911	0.69675	0.90425
2-Hydroxybutyric acid	0.041292	0.37195	0.7109	0.90425
Homoserine	0.039607	0.35675	0.72221	0.90425
Leucine	-0.038557	-0.34727	0.72929	0.90425
Tryptophan	0.037895	0.3413	0.73376	0.90425
Gluconic acid	0.036951	0.33279	0.74015	0.90425
Ornithine	0.035674	0.32127	0.74883	0.90425
Alanine	0.031103	0.28006	0.78014	0.91498
Choline	-0.030209	-0.272	0.78631	0.91498
Serine	-0.025848	-0.23271	0.81657	0.93323
Glutamic acid	-0.019584	-0.17629	0.86051	0.95323
Proline	-0.019108	-0.172	0.86386	0.95323
Adenosine diphosphate (ADP)	-0.011021	-0.099196	0.92123	0.99315
Valine	-0.0084686	-0.07622	0.93943	0.99315
Tyrosine	-0.0074647	-0.067185	0.9466	0.99315
Glycine	0.0043594	0.039235	0.9688	0.99402
Guanosine diphosphate (GDP)	0.0013403	0.012063	0.99041	0.99402
Threonine	-0.00083565	-0.0075209	0.99402	0.99402

Table shows metabolites ranked in order of decreasing significance based on absolute FDR values. FDR, adjusted p-value (False discovery rate corrected).
