# Supplementary material for: Schistosoma haematobium infection is associated with alterations in energy and purine-related metabolism in preschool-aged children
Source: PLoS Negl Trop Dis. 2020 Dec 14;14(12):e0008866. doi: 10.1371/journal.pntd.0008866 (PMC7735607; doi:10.1371/journal.pntd.0008866)
Supplement: S4 Table — (PDF) [file pntd.0008866.s010.pdf]

S4 Table: Output from OPLSDA model S-plot for sex, along with corresponding VIP values

Metabolite	p[1]	p(corr)[1]	VIP
Creatinine	0.27549	0.83449	2.8572
Citrulline	0.28376	0.74286	3.0786
Sarcosine	0.2158	0.58758	1.6958
GABA	0.24761	0.51792	2.4472
cis-Aconitic acid	0.24515	0.48237	2.724
β-Alanine	0.12627	0.45482	0.70673
Citric acid	0.1852	0.44703	0.84383
Ribulose-5-phosphate	0.12263	0.44449	0.82369
Isocitric acid	0.21912	0.39771	2.1385
Inosine	0.12335	0.39247	0.78589
Gluconic acid	0.091583	0.38912	0.14954
Glyoxylic acid	0.091254	0.38088	0.53769
Hydroxyproline	0.12839	0.37893	0.59623
Malic acid	0.089404	0.37613	0.32553
Guanosine monophosphate (GMP)	0.18069	0.36823	1.185
Lactic acid	0.15143	0.3672	0.3541
Tyrosine	0.10023	0.3661	0.035139
Threonine	0.10289	0.36533	0.0040469
Spermidine	0.078093	0.35967	0.34483
3-Phosphoglyceric acid	0.106	0.35216	0.96721
Alanine	0.098844	0.34014	0.15541
Valine	0.090363	0.33706	0.039038
Ornithine	0.10801	0.32348	0.20481
Methionine	0.10469	0.31104	0.55285
Histidine	0.076237	0.30659	0.23536
2-Oxoisovaleric-acid	0.20386	0.30528	2.6467
Glycine	0.080119	0.29608	0.020284
N.N-Dimethylglycine	0.10961	0.29401	0.75786
Glucose-1-phosphate	0.080837	0.29223	1.0281
Creatine	0.085623	0.28529	0.29888
Proline	0.083795	0.27386	0.10053
Inosine monophosphate (IMP)	0.10985	0.27044	0.88282
Pyruvic acid	0.11333	0.25751	0.9147
Glutamine	0.10691	0.25725	0.64903
Tryptophan	0.082756	0.24162	0.22317
Leucine	0.060968	0.24067	0.16795
Ethanolamine phosphate	0.096447	0.22581	0.44797
Glutamic acid	0.077948	0.22498	0.11667
Carnosine	0.077743	0.22476	0.25821
Adenosine monophosphate (AMP)	0.10243	0.21539	0.84452
2-Phosphoglyceric acid	0.049194	0.2145	0.55225
Lysine	0.067508	0.21384	0.50823
Glucose-6-phosphate	0.11745	0.2108	0.99899
Fumaric-acid	0.058516	0.1974	0.84955

Metabolite	p[1]	p(corr)[1]	VIP
Serine	0.058128	0.1968	0.13128
Betaine	0.071743	0.18678	0.49792
Uracil	0.038722	0.18235	0.34907
Isoleucine	0.050402	0.15271	0.38322
Choline	0.040908	0.15246	0.13937
Aspartic acid	0.034616	0.12663	0.42621
Homoserine	0.050824	0.11895	0.29098
Uridine diphosphate (UDP)	0.024667	0.11412	0.38436
Uridine	0.041775	0.10853	0.29927
2-Hydroxybutyric acid	0.031232	0.10034	0.221
Phenylalanine	0.029308	0.093128	0.469
3-Hydroxybutyric.acid	0.029264	0.080126	0.65323
Adenosine diphosphate (ADP)	0.032083	0.078068	0.077878
Guanine	0.015178	0.070223	0.38436
Succinic acid	0.021835	0.063789	0.37276
Arginine	0.0083901	0.019567	0.70061
2-Oxoglutaric acid	0.0085815	0.015868	0.45414
Hypoxanthine	0.0035884	0.0089486	0.59623
Guanosine diphosphate (GDP)	-0.0044877	-0.012625	0.0081925
Asparagine	-0.048699	-0.10995	1.407

Table shows metabolites ranked in order of decreasing correlation or reliability coefficient [p(corr)]. p[1], covariance or contributions; p(corr)[1], correlation or reliability coefficient; VIP, variable importance in the projection.
