# Supplementary material for: Schistosoma haematobium infection is associated with alterations in energy and purine-related metabolism in preschool-aged children
Source: PLoS Negl Trop Dis. 2020 Dec 14;14(12):e0008866. doi: 10.1371/journal.pntd.0008866 (PMC7735607; doi:10.1371/journal.pntd.0008866)
Supplement: S5 Table — (PDF) [file pntd.0008866.s011.pdf]

S5 Table: Follow-up MANOVA output for influence of infection on metabolite profiles

Variable	F value	Hypothesis/Total df	Error df	Partial Eta-squared	p-value
Intercept	0	56	21	0	1.000
Age	1.432	56	21	0.792	0.184
Sex	0.742	56	21	0.664	0.813
Infection status	2.231	56	21	0.856	0.023
Age * Sex	0.960	56	21	0.719	0.567
Sex * Infection status	1.901	56	21	0.835	0.053
Age * Infection status	1.056	56	21	0.738	0.463
Equation: Intercept + Age + Sex + Infection status+ Age * Sex + Sex * Infection status + Age * Infection status					
	**Model to obtain residuals**				
Intercept	0	56	24	0	1.000
Age	1.214	56	24	0.739	0.307
Sex	0.778	56	24	0.645	0.782
Age * Sex	1.002	56	24	0.700	0.516
Equation: Intercept + Age + Sex +Age * Sex					

df, degrees of freedom.
