# Supplementary material for: Schistosoma haematobium infection is associated with alterations in energy and purine-related metabolism in preschool-aged children
Source: PLoS Negl Trop Dis. 2020 Dec 14;14(12):e0008866. doi: 10.1371/journal.pntd.0008866 (PMC7735607; doi:10.1371/journal.pntd.0008866)
Supplement: S6 Table — (PDF) [file pntd.0008866.s012.pdf]

S6 Table: Important metabolites identified from fold change analysis by infection status

Metabolite	Fold Change (FC)	log_2 (FC)
Choline	8.8175	3.1404
Lactic acid	6.1847	2.6287
Glutamic acid	2.4991	1.3214
Serine	2.4807	1.3107
Succinic acid	2.4599	1.2986
Histidine	2.453	1.2946
Phenylalanine	2.3656	1.2422
Lysine	2.3145	1.2107
Pyruvic acid	2.2875	1.1937
Hypoxanthine	2.1134	1.0796
Ornithine	2.1105	1.0776
Leucine	2.1069	1.0752
Homoserine	2.0195	1.014
Glucose-6-phosphate	0.48149	-1.0544
Spermidine	0.4527	-1.1434
Asparagine	0.44836	-1.1573
2-Hydroxybutyric acid	0.44462	-1.1694
Inosine monophosphate (IMP)	0.42322	-1.2405
Ethanolamine phosphate	0.41315	-1.2752
Guanosine monophosphate (GMP)	0.32008	-1.6435
Adenosine monophosphate (AMP)	0.25	-2.000
Adenosine diphosphate (ADP)	0.1844	-2.4391
Sarcosine	0.17928	-2.4797
Inosine	0.13699	-2.8679
3-Phosphoglyceric acid	0.1361	-2.8773

Table shows metabolites ranked in order of decreasing absolute fold change. Analysis is according to uninfected/infected ratio.
