# Supplementary material for: Schistosoma haematobium infection is associated with alterations in energy and purine-related metabolism in preschool-aged children
Source: PLoS Negl Trop Dis. 2020 Dec 14;14(12):e0008866. doi: 10.1371/journal.pntd.0008866 (PMC7735607; doi:10.1371/journal.pntd.0008866)
Supplement: S7 Table — (PDF) [file pntd.0008866.s013.pdf]

S7 Table: Correlation pattern analysis of compounds showing patterns from negative to positive for infection

Metabolite	Correlation	t-statistic	p-value	FDR
Adenosine diphosphate (ADP)	0.43076	4.2959	4.80E-05	0.002689
3-Phosphoglyceric acid	0.4095	4.0397	0.00012098	0.0033875
Adenosine monophosphate (AMP)	0.36253	3.5009	0.00075695	0.01413
Inosine	0.27435	2.5677	0.012077	0.11995
Asparagine	0.26342	2.4575	0.016124	0.11995
Lactic acid	-0.26245	-2.4479	0.016532	0.11995
Choline	-0.26204	-2.4438	0.016708	0.11995
Serine	-0.2554	-2.3774	0.019791	0.11995
2-Hydroxybutyric acid	0.25256	2.3492	0.021249	0.11995
cis-Aconitic acid	-0.25007	-2.3245	0.022602	0.11995
Sarcosine	0.24839	2.3078	0.023561	0.11995
Histidine	-0.23692	-2.1948	0.031042	0.13583
Guanosine monophosphate (GMP)	0.23626	2.1882	0.031533	0.13583
Glucose-6-phosphate	0.22272	2.0561	0.042991	0.15628
Ethanolamine phosphate	0.22243	2.0533	0.04327	0.15628
Glutamic acid	-0.22102	-2.0396	0.044652	0.15628
Creatinine	-0.2157	-1.9881	0.050184	0.1587
Succinic acid	-0.21244	-1.9566	0.053837	0.1587
Alanine	-0.21244	-1.9566	0.053844	0.1587
Glycine	-0.19987	-1.8358	0.070053	0.19615
Lysine	-0.19299	-1.7702	0.080451	0.21213
Leucine	-0.19122	-1.7533	0.083335	0.21213
Phenylalanine	-0.18497	-1.694	0.094112	0.22914
Aspartic acid	-0.17888	-1.6363	0.10566	0.24654
Ornithine	-0.17441	-1.5941	0.11481	0.25718
Threonine	-0.17199	-1.5713	0.12001	0.25848
Tyrosine	-0.13279	-1.2058	0.23141	0.46351
Inosine monophosphate (IMP)	0.13183	1.1969	0.23484	0.46351
Tryptophan	0.12799	1.1614	0.24888	0.46351
2-Oxoisovaleric acid	0.12791	1.1607	0.24917	0.46351
Spermidine	0.12594	1.1426	0.25658	0.46351
Pyruvic acid	-0.1144	-1.0364	0.30308	0.53039
Creatine	0.10995	0.99558	0.32242	0.53498
Citrulline	-0.10941	-0.99064	0.32481	0.53498
2-Oxoglutaric acid	-0.087052	-0.78646	0.43389	0.69423
Hypoxanthine	-0.084189	-0.7604	0.44922	0.69879
Isoleucine	-0.075729	-0.68353	0.49623	0.75104
Gamma aminobutyric acid (GABA)	-0.072465	-0.65391	0.51503	0.75406
Carnosine	0.069942	0.63102	0.5298	0.75406
Homoserine	-0.068454	-0.61753	0.53862	0.75406
Hydroxyproline	0.060526	0.54574	0.58675	0.78979

Metabolite	Correlation	t-statistic	p-value	FDR
Glutamine	0.059074	0.5326	0.59577	0.78979
Valine	-0.057369	-0.51717	0.60645	0.78979
3-Hydroxybutyric acid	0.052369	0.47197	0.63822	0.81228
Gluconic acid	-0.042401	-0.38196	0.70349	0.86895
Malic acid	-0.040099	-0.36118	0.7189	0.86895
Uridine	-0.038359	-0.34548	0.73063	0.86895
Ribulose-5-phosphate	0.033535	0.30198	0.76344	0.86895
Proline	-0.033532	-0.30196	0.76345	0.86895
Arginine	-0.031727	-0.28568	0.77585	0.86895
Methionine	-0.029174	-0.26267	0.79347	0.87126
Citric acid	0.026801	0.2413	0.80993	0.87223
N.N-Dimethylglycine	-0.023034	-0.20736	0.83625	0.88358
Isocitric acid	0.015931	0.1434	0.88633	0.91916
Betaine	-0.0096291	-0.086666	0.93115	0.93889
β-Alanine	0.0085451	0.076909	0.93889	0.93889

Table shows metabolites ranked in order of decreasing significance based on absolute FDR values. FDR, adjusted p-value (False discovery rate corrected).
