# Supplementary material for: Schistosoma haematobium infection is associated with alterations in energy and purine-related metabolism in preschool-aged children
Source: PLoS Negl Trop Dis. 2020 Dec 14;14(12):e0008866. doi: 10.1371/journal.pntd.0008866 (PMC7735607; doi:10.1371/journal.pntd.0008866)
Supplement: S8 Table — (PDF) [file pntd.0008866.s014.pdf]

S8 Table: Output from OPLSDA model S-plot for infection, along with corresponding VIP values

Metabolite	p[1]	p(corr)[1]	VIP
Adenosine monophosphate (AMP)	0.16332	0.71903	1.5507
3-Phosphoglyceric acid	0.13835	0.71135	1.4998
Adenosine diphosphate (ADP)	0.17779	0.62789	2.297
Ethanolamine phosphate	0.19096	0.5937	1.3473
Glucose 6 phosphate	0.2032	0.55796	1.5275
Guanosine monophosphate (GMP)	0.16846	0.51693	1.4499
Inosine monophosphate (IMP)	0.11625	0.3841	0.75132
Asparagine	0.13774	0.32756	2.086
Sarcosine	0.06345	0.31531	0.94127
Carnosine	0.094955	0.31492	0.39715
Spermidine	0.053364	0.29941	0.4227
2-Hydroxybutyric acid	0.09136	0.27153	1.6003
Glutamine	0.10048	0.26156	0.42738
Inosine	0.056996	0.25414	1.1587
Creatine	0.079849	0.24129	0.68521
Ribulose-5-phosphate	0.048322	0.20786	0.14681
2-Oxoisovaleric acid	0.074067	0.17949	0.99399
Isocitric acid	0.068813	0.17527	0.11779
Hydroxyproline	0.043562	0.14883	0.33362
Tryptophan	0.058044	0.14599	0.95828
Citric acid	0.053018	0.12601	0.21236
β-Alanine	0.026035	0.10311	0.040634
N.N-Dimethylglycine	0.025649	0.093055	0.11956
Proline	0.01185	0.036949	0.20252
Betaine	0.011335	0.031553	0.065143
3-Hydroxybutyric acid	0.0062571	0.024438	0.25251
Pyruvic acid	0.0044649	0.014935	0.64409
Malic acid	-0.0055014	-0.025446	0.16326
Citrulline	-0.0087832	-0.026973	0.67094
cis-Aconitic acid	-0.010699	-0.028551	1.7647
gamma-aminobutyric acid (GABA)	-0.022744	-0.05116	0.60668
Methionine	-0.018806	-0.05124	0.20164
Gluconic acid	-0.017143	-0.071042	0.19269
Uridine	-0.026073	-0.079352	0.23735
Isoleucine	-0.030496	-0.090193	0.4822
Homoserine	-0.02843	-0.10138	0.36151
Arginine	-0.037684	-0.11043	0.20389
Valine	-0.035022	-0.12602	0.30024
Lactic acid	-0.044449	-0.12925	1.6997
Hypoxanthine	-0.052029	-0.12984	0.63531
Tyrosine	-0.049312	-0.17242	0.7152
2-Oxoglutaric acid	-0.049913	-0.18307	0.44697

Metabolite	p[1]	p(corr)[1]	VIP
Succinic acid	-0.066637	-0.18646	1.4298
Asp	-0.046762	-0.20107	0.78341
Creatinine	-0.046943	-0.20107	0.94836
Threonine	-0.069166	-0.24389	0.91853
Ornithine	-0.08025	-0.25436	1.0362
Glycine	-0.06732	-0.25854	0.98005
Alanine	-0.070685	-0.26489	1.0675
Phenylalanine	-0.080367	-0.29783	0.93998
Leucine	-0.085806	-0.32052	0.964
Lysine	-0.09314	-0.32526	1.0408
Glutamic acid	-0.11037	-0.33346	1.3776
Histidine	-0.09129	-0.35142	1.159
Serine	-0.10134	-0.36012	1.3535
Choline	-0.12508	-0.40257	1.5333

Table shows metabolites ranked in order of decreasing correlation or reliability coefficient [p(corr)]. p[1], covariance or contributions; p(corr)[1], correlation or reliability coefficient; VIP, variable importance in the projection.
