# Supplementary material for: Schistosoma haematobium infection is associated with alterations in energy and purine-related metabolism in preschool-aged children
Source: PLoS Negl Trop Dis. 2020 Dec 14;14(12):e0008866. doi: 10.1371/journal.pntd.0008866 (PMC7735607; doi:10.1371/journal.pntd.0008866)
Supplement: S9 Table — (PDF) [file pntd.0008866.s015.pdf]

S9 Table: MANOVA output for influence of infection intensity on metabolite profiles

Variable	F value	Hypothesis/ Total df	Error df	Partial Eta-squared	p-value
	**Model with all metabolites (n=56)**				
Intercept	0.309	56	26	0.399	1.000
Infection intensity (log transformed egg count +1)	2.729	56	26	0.855	0.003
Equation: Intercept + Infection intensity (log (x+1) transformed)					
	**Model using data for significant metabolites from OPLSDA for infection (n=4)**				
Intercept	0.586	4	78	0.29	0.674
Infection intensity (log transformed egg count +1)	5.178	4	78	0.21	0.001
Equation: Intercept + Infection intensity (log (x+1) transformed)					

df, degrees of freedom.
