# Supplementary material for: Schistosoma haematobium infection is associated with alterations in energy and purine-related metabolism in preschool-aged children
Source: PLoS Negl Trop Dis. 2020 Dec 14;14(12):e0008866. doi: 10.1371/journal.pntd.0008866 (PMC7735607; doi:10.1371/journal.pntd.0008866)
Supplement: S10 Table — (PDF) [file pntd.0008866.s016.pdf]

S10 Table: Correlation pattern analysis of metabolites showing patterns from positive for infection to negative post-treatment

Metabolite	Correlation	t-statistic	p-value	FDR
Glucose-6-phosphate	-0.48165	-1.738	0.11285	0.24623
3-Phosphoglyceric acid	-0.47	-1.6839	0.12312	0.24623
Adenosine diphosphate (ADP)	-0.39272	-1.3504	0.20666	0.27554
Adenosine monophosphate (AMP)	-0.25002	-0.81657	0.4332	0.4332

Table shows metabolites ranked in order of decreasing significance based on absolute FDR values. FDR, adjusted p-value (False discovery rate corrected).
