# Supplementary material for: Schistosoma haematobium infection is associated with alterations in energy and purine-related metabolism in preschool-aged children
Source: PLoS Negl Trop Dis. 2020 Dec 14;14(12):e0008866. doi: 10.1371/journal.pntd.0008866 (PMC7735607; doi:10.1371/journal.pntd.0008866)
Supplement: S11 Table — (PDF) [file pntd.0008866.s017.pdf]

S11 Table: Analysis output from metabolite pathway analysis

Metabolic pathway	Total Cmpds	Hits	Raw p	-log(p)	Holm adjust	FDR	Impact
Purine metabolism	92	2	3.61E-05	10.229	0.000253	0.000253	0.06428
Nitrogen metabolism	39	1	0.000757	7.1862	0.004542	0.002650	0
Glycolysis or Gluconeogenesis	31	1	0.042991	3.1468	0.21496	0.042991	0.06977
Pentose phosphate pathway	32	1	0.042991	3.1468	0.21496	0.042991	0.04348
Starch and sucrose metabolism	50	1	0.042991	3.1468	0.21496	0.042991	0.02439
Galactose metabolism	41	1	0.042991	3.1468	0.21496	0.042991	0.01724
Amino sugar and nucleotide sugar metabolism	88	1	0.042991	3.1468	0.21496	0.042991	0.00917

Table shows metabolites ranked in order of decreasing significance based on absolute FDR values. FDR, adjusted p-value (False discovery rate correction for multiple comparisons); Holm adjust, adjusted p value (Holm-Bonferroni correction for multiple comparisons); Raw p, unadjusted p values; Hits, number of metabolite hits from test data found in pathway, Total Cmpds, total number of compounds in pathway.
